# Treatment of preschool children presenting to the emergency department with wheeze with azithromycin: A placebo-controlled randomized trial

**DOI:** 10.1371/journal.pone.0182411

**Published:** 2017-08-03

**Authors:** Piush J. Mandhane, Patricia Paredes Zambrano de Silbernagel, Yin Nwe Aung, Janie Williamson, Bonita E. Lee, Sheldon Spier, Mary Noseworthy, William R. Craig, David W. Johnson

**Affiliations:** 1 Department of Pediatrics, Faculty of Medicine and Dentistry, University of Alberta, Edmonton, Alberta, Canada; 2 Department of Community Medicine, Faculty of Medicine and Health Sciences, UCSI University, Kuala Lumpur, Malaysia; 3 Department of Pediatrics, Cumming School of Medicine, University of Calgary, Calgary, Alberta, Canada; 4 Departments of Pediatrics, Emergency Medicine and Physiology and Pharmacology, Cumming School of Medicine, Alberta Children’s Hospital Research Institute, University of Calgary, Calgary, Alberta, Canada; Universite de Bretagne Occidentale, FRANCE

## Abstract

**Background:**

Antibiotics are frequently used to treat wheezing children. Macrolides may be effective in treating bronchiolitis and asthma.

**Method:**

We completed a prospective, double-blinded, randomized placebo-control trial of azithromycin among pre-school children (12 to 60 months of age) presenting to the emergency department with wheeze. Patients were randomized to receive either five days of azithromycin or placebo. Primary outcome was time to resolution of respiratory symptoms after treatment initiation. Secondary outcomes included the number of days children used a Short-Acting Beta-Agonists during the 21 day follow-up and time to disease exacerbation during the following six months (unscheduled health care visit or treatment with an oral corticosteroid for acute respiratory symptoms).

**Results:**

Of the 300 wheezing children recruited, 222 and 169 were analyzed for the primary and secondary outcomes, respectively. The treatment groups had similar demographics and clinical parameters at baseline. Median time to resolution of respiratory symptoms was four days for both treatment arms (interquartile range (IQR) 3,6; p = 0.28). Median number of days of Short-Acting Beta-Agonist use among those who received azithromycin was four and a half days (IQR 2, 7) and five days (IQR 2, 9; p = 0.22) among those who received placebo. Participants who received azithromycin had a 0.91 hazard ratio for time to six-month exacerbation compared to placebo (95% CI 0.61, 1.36, p = 0.65). A pre-determined subgroup analysis showed no differences in outcomes for children with their first or repeat episode of wheezing. There was no significant difference in the proportion of participants experiencing an adverse event.

**Conclusion:**

Azithromycin neither reduced duration of respiratory symptoms nor time to respiratory exacerbation in the following six months after treatment among wheezing preschool children presenting to an emergency department. There was no significant effect among children with either first-time or prior wheezing.

## Background

Preschool wheeze represents significant morbidity for the individual and a significant burden to society. One in three children has an episode of wheezing prior to their third birthday; almost 50% of children wheeze by six years of age[1,2]. While recent evidence suggests overall emergency department visits for asthma have decreased, visits for children with wheeze in the first four years of life have not[3]. Management of pre-school children who wheeze is controversial, with only supplemental oxygen, hydration and inhaled short acting β_2_-agonists being widely accepted[4,5].

A handful of recently published studies have investigated whether macrolide antibiotics are effective at reducing the duration of symptoms in infants with bronchiolitis and preschool-aged children with recurrent wheeze[6–9]. There are several potential pathophysiological rationale for the use of macrolides in these populations of young children. The first is that recent studies suggest that bacteria or the co-infection of virus and bacteria may be present during wheezing episodes in children[10,11]. Carlsson et. al.[10] identified the coinfection of virus and bacteria in 55% of acute wheezing episodes among children under three years of age. Kloepfer et. al.[11] reported an increased detection of specific bacterial pathogens during rhinovirus infection in children (four to 12 years of age) with and without asthma diagnosis. As a result, macrolides’ antibacterial activit*y* may result in quicker resolution as compared with no antibiotic treatment[7,9,10,12,13].

The second rationale is that macrolides are widely recognized to have immune-modulatory and potential antiviral properties[14]. The efficacy of azithromycin in the absence of bacterial infection emphasizes the anti-inflammatory and potential antiviral properties of azithromycin[10]. Beigelman et. al.[13] observed a decrease in upper airway (nasal lavage) IL-8 levels after the administration of azithromycin for 14 days to infants with respiratory syncytial virus bronchiolitis. In eosinophilic asthmatics, clarithromycin improved symptoms and had a reduction of blood eosinophil and eosinophil cationic protein levels on serum and sputum[15]. Clarithromycin significantly reduced airway concentrations of interleukin 8 (IL-8), metalloproteinase 9 (MMP-9), neutrophil elastase, neutrophil numbers, in a randomized control trial of adults with severe, refractory asthma[16].

Although several recently published studies in infants with bronchiolitis treated with macrolides did not show a benefit[6,7,13,17], other studies have shown that the early administration of azithromycin in pre-school children with multiple recurrent wheezing episodes reduced the progression to or severity of lower respiratory infection[8,9]. We hypothesized that treatment of pre-school children 12 to 60 months of age visiting the emergency department, regardless of the severity or chronicity of previous wheezing episodes, with acute wheezing with five days of azithromycin would reduce the duration of their respiratory symptoms compared with children treated with placebo.

## Methods

### Trial design and participants

We completed a prospective, double-blinded, placebo-control randomized trial of azithromycin among wheezy pre-school children (12 to 60 months of age) who presented to the Alberta Children’s and Stollery Children’s Hospitals’ emergency departments, between January 2011 to May 2014 (**S1 File** presents the study design). Children were excluded if they used antibiotics in the past 30 days, had a contraindication to the use of a macrolide, significant medical co-morbidities, a language barrier, or would not be able to complete follow-up (**Table 1**presents the study inclusion and exclusion criteria). Analysis was performed following the intention-to-treat principle. Informed consent was obtained from the parent or legal caretaker. The research ethics boards of both the University of Alberta and University of Calgary approved the study. Trial Registry: www.clinicaltrials.gov; Identifier: NCT01008761.

**Table 1:** Study inclusion and exclusion criteria.

Inclusion criteria	
	12–60 months of age
	Presented to one of the participating EDs
	Wheezing on physical exam (noted by physician or nurse)
Exclusion criteria	
	Use of antibiotics during the 30 days previous to the study
	Contraindication for use of macrolides
	Significant medical co-morbidities
	Current enrolment in another study, or enrolment within four weeks previous to the study
	Language barrier
	No access to a telephone
	Not available to complete follow-up

### Intervention

Patients were randomized to receive either five days of azithromycin (obtained from either PMS or Pfizer depending on availability) or placebo (produced by the Drug Development and Innovation Centre at the University of Alberta). Subjects were given orally ten mg/kg for day one and five mg/kg/day for four days. Each bottle contained sufficient drug to adequately dose children who weigh up to 30 kg (above the 95 percentile weight for 60 month old children). Azithromycin and placebo powders were mixed with a fixed volume of sterile water, and were indistinguishable by appearance, smell or taste.

### Primary outcome

A calendar diary[18] (**S1 File**) was used to collect daily respiratory symptoms. Parents were asked to place different coloured stickers on the calendar corresponding to their child’s respiratory symptoms. The primary outcome was the time (days) to respiratory symptoms resolution defined as three consecutive days of their child having either no or usual respiratory symptoms on the calendar diary[18].

### Secondary outcomes

Secondary outcomes included 1) short-acting beta agonist use and 2) time to disease exacerbation. Only children prescribed a Short-Acting Beta Agonists at the time of discharge from the emergency department were included in the secondary outcome analysis. Among study participants prescribed salbutamol (Airomir™, 100 μg per puff) in the emergency department, the number of days the child used salbutamol during the 21 days following study enrolment was assessed by dose counter (Doser™, Meditracker). Time to disease exacerbation was assessed by questionnaire administered at days 35, 63, 105, 147 and 189 (**Table 2 and S1 File**). Disease exacerbation was defined as either an unscheduled visit to a physician/nurse practitioner or treatment with an oral corticosteroid for acute respiratory symptoms (cough, wheeze, or respiratory distress based on parental perception) that occurred after resolution of the child’s initial symptoms.

**Table 2:** Demographics of children included in the primary analysis.

Demographics	Azithromycin (N = 110)	Placebo (N = 112)
Male (n (%))	77 (70.0)	83 (74.1)
Age in months–mean (SD)	34.8 (13.6)	30.5 (13.9)
Cough at triage (n (%))	109 (99.1)	108 (96.4)
Inhalers used prior ED (n (%))		
SABA	69 (62.7)	66 (58.9)
Inhaled Corticosteroids	39 (35.5)	41 (36.6)
Diagnosis of allergies/eczema (n (%))	47 (42.7)	46 (41.1)
Family history of atopy (n (%))	69 (62.7)	66 (58.9)
Smokers in household (n (%))	26 (23.6)	20 (17.9)
Attend day care (n (%))	49 (44.6)	53 (47.3)
Ever wheeze before	97 (88.2)	98 (87.5)
Ever diagnosed asthma by MD	45 (40.9)	39 (34.8)
Ever had any treatment for asthma or wheezing	74 (67.3)	84 (75.0)
Prior ED visit for asthma or wheezing	91 (82.7)	97 (86.6)
Prior hospitalization for asthma or wheezing	36 (32.7)	27 (24.1)

Data given as means (SD) or numbers (%).

### Sample size

Sample size calculations were based on a previous report in patients with an asthma exacerbation treated with a macrolide versus placebo, whose symptoms resolved 50% more rapidly, and our estimation that a 50% faster resolution of symptoms equated to 1.36 days[19]. To detect this difference in days to resolution, we determined we needed to enroll 440 subjects (110 children with first time wheeze and 330 with previous wheeze) with 80% power at the 0.05 level, a standard deviation of 6 days in symptoms duration, and a 10% loss to follow-up.

### Randomization

The Drug Development and Innovation Center generated the random allocation sequence using random-number generating software (2 x 2 variable block with four to eight per block stratified by study site and history of wheeze (first versus previous episode of wheeze). The allocation sequence was concealed from research assistants, investigators and participants. The research assistant in the emergency department enrolled participants with assignment to intervention based on the randomization sequence and wheezing history. Each child was assigned an order number and received the study drug in the corresponding pre-packed bottle.

### Blinding

All investigators, research assistants, and participants were masked to allocation of treatment. Participants, study personnel, study investigators and data analysts were blinded to allocation group.

### Data collection

Parents completed a baseline questionnaire (**Table 2 and S1 File**) about the child’s past medical history, previous episodes of respiratory symptoms, asthma and asthma medication use, parental history of atopy, and home environment. The adverse event questionnaire, continued until day 35 (**Table 2**), was completed if the child had symptoms believed to be related to the study drug (e.g. gastro-intestinal symptoms, headache, rash, hives, irregular heart rate). Skin prick testing, to assess for atopy, was completed on day 21 at an in-clinic visit[18] (**S1 File**). After discharge, the research assistant collected information related to the vital signs at discharge, the medication provided by the attendant physician in the emergency department, the medicine prescribed to be used at home (short-acting beta agonist, oral steroid or inhaled corticosteroids), the final diagnosis, and whether patients were discharged or hospitalized (**S1 File**).

## Statistical methods

### Time to symptom resolution (primary outcome)

The time to symptom resolution between treatment groups was compared using Mann-Whitney U test. We also conducted pre-specified subgroup analyses between treatment groups by type of wheeze (first time vs. previous). Children were defined as having first-time wheeze if their current wheezing episode had lasted for less than one month and they had never previously wheezed prior to this episode. The data are presented as median and interquartile range (IQR). Interaction between treatment effect and a number of baseline characteristics was assessed using Poisson regression.

### Short-acting beta-agonists use (secondary outcome)

The number of days children used a Short-Acting Beta Agonists inhaler at least one time in the drug and placebo groups were compared using Mann-Whitney U test. The data are presented as medians and interquartile range (IQR).

### Time to exacerbation (secondary outcome)

Survival rates are expressed as the percentage days free of disease exacerbation calculated using Kaplan-Meier methods. Kaplan-Meier curves comparing azithromycin and placebo are shown up to 189 days follow-up (participants with no exacerbation were censored at this time). Cox proportional hazards model was used to calculate the hazards ratios and 95% confidence intervals between treatment groups. Analyses were carried out in Stata version 13.

## Results

### Study participants

**Fig 1:**
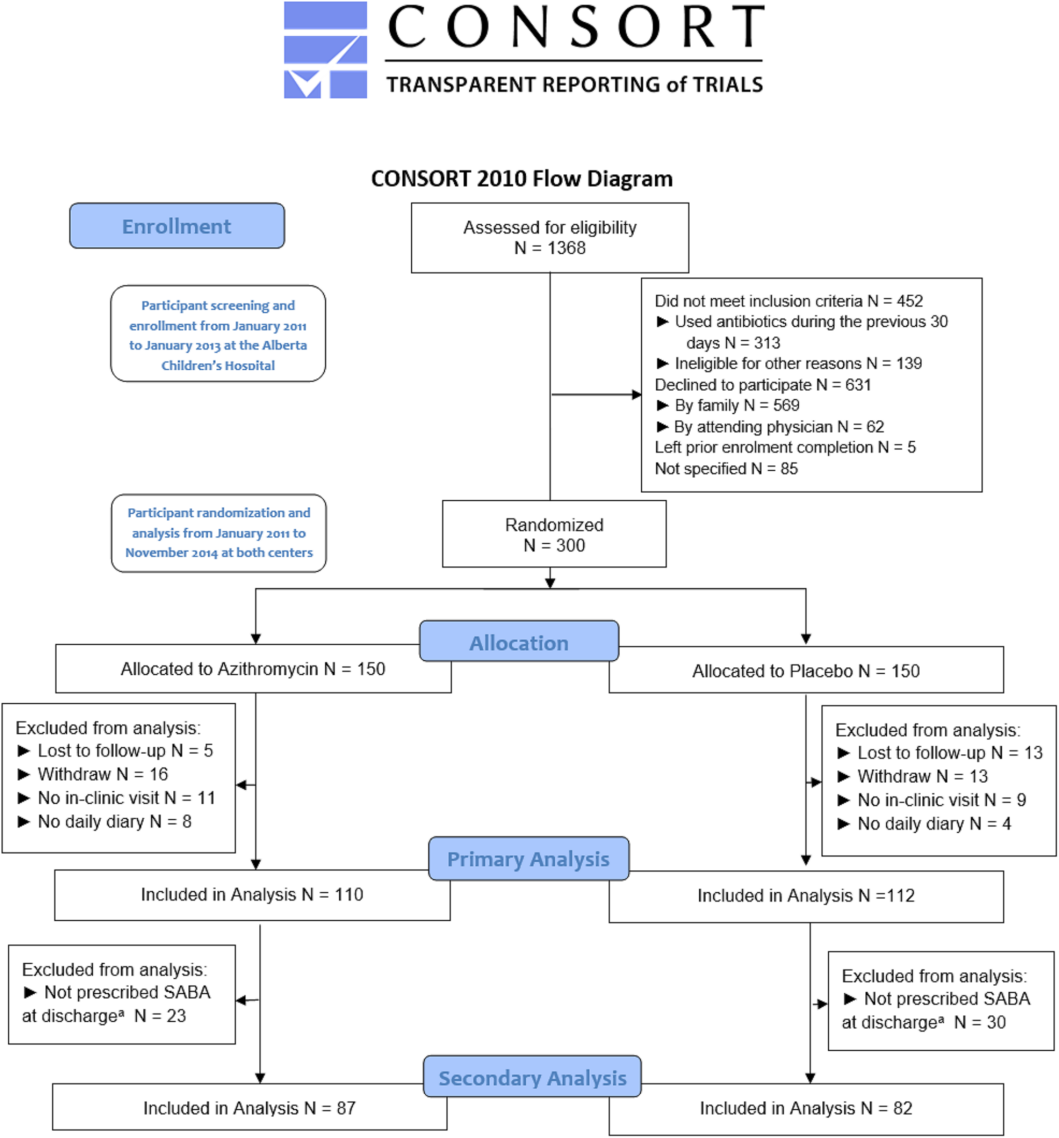
Study participant disposition: Participant screening and enrollment from January 2011 to January 2013 at the Alberta Children’s Hospital, participant randomization and analysis throughout the study and centers. CONSORT flow diagram of the study enrolment for pre-school wheezing children. ^a^ Prescription of Short-Acting Beta Agonist (SABA) at discharge was inclusion criteria for secondary analysis.

A total of 1368 children between 12 and 60 months of age with wheeze were assessed for eligibility at The Alberta Children’s Hospital from January 2011 to January 2013. Thirty-three percent of children (453/1368) did not meet eligibility criteria with the majority excluded (69%; 313/453) because they had received antibiotics within the prior 30 days. Of the 915 eligible children, 62% (569/915) of families declined to participate in the study, 62 children’s (7%) emergency department attending physician refused inclusion of these children in the study, and 5 (0.5%) children left the emergency department with their parents before enrolment completion (**Fig 1**). The number of eligible children at the Stollery Children’s Hospital was not tracked.

A total of 300 wheezing children were enrolled in the study between January 2011 and May 2014 (150 children into the azithromycin and 150 children into placebo arms) with follow-up ending November 2014. The Alberta Children’s Hospital enrolled 195 children and the Stollery Children’s Hospital enrolled 105 children. The study was terminated due to exhaustion of grant funding. In total, 222 families (74%) returned the daily diary (n = 110 who received azithromycin & n = 112 who received placebo) and were included in the primary analysis. Of the 78 children excluded from primary analysis (**Fig 1**): 31 families (40%) did not return a completed daily diary, 29 families (37%) withdrew consent to participate in the study and 18 children (23%) were lost to follow-up. Participants not included in the analysis had similar age, gender, and previous wheezing history compared to those children included in the analysis (**S1 File**). For the secondary outcome analysis, 169 eligible children were included. As per protocol, 53 children were excluded from this analysis because they were not prescribed salbutamol at discharge from the emergency department. Over 93% of participants (279/300) provided adverse event information.

Demographic characteristics were similar between treatment arms. Participants were mostly male (over 70% for both group; **Table 2**) with a mean age at recruitment in the azithromycin group of 34.8 months (SD: 13.6) and 30.5 months (SD: 13.9) in the placebo group. Sixty-four (29%) participants fulfilled the definition for first time wheeze at enrolment. Inhaled corticosteroids were used with 35.5% participants in the azithromycin group and 36.6% participant in the placebo group (**Table 2**). Almost 60% of participants in both treatment groups had a family history of atopy. Additional demographic data and demographic differences for children with first and prior wheeze are presented in **Table 2**and in the online supplement in **S1 File**.”

### Time to respiratory symptom resolution (primary outcome)

**Fig 2:**
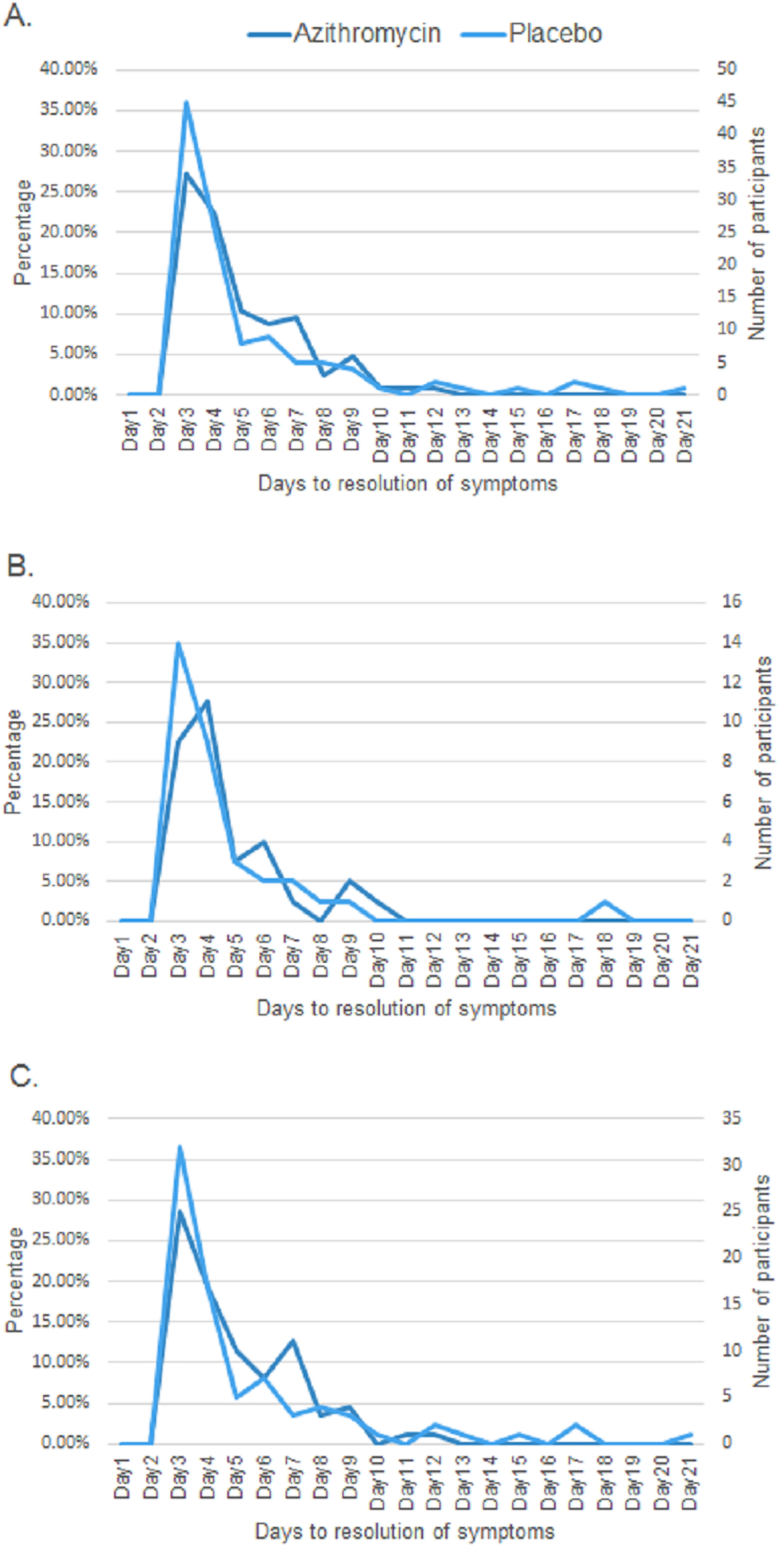
Time to symptoms resolution (days) by allocation. **(A)**, first-time wheezers **(B)**, and previous wheezers **(C)**. There was no significant difference between pre-school children randomized to azithromycin and placebo.

The time to resolution of symptoms was not normally distributed among groups (Shapiro-Wilk’s p<0.05) (**S1 File**). The median time to respiratory symptoms resolution was four days for both groups (IQR 3, 6) and was not statistically different between treatment groups (p = 0.28; **S1 File**). Similarly, there was no significant difference in time to resolution by prior wheezing status. Among those with first-time wheeze, the median time to resolution of symptoms was four days (IQR 3,6; **Fig 2**) for the azithromycin group and four days (IQR 3,5; p = 0.40; **S1 File**) for the placebo group. Among those with previous wheeze, the median time to resolution of symptoms was four days (IQR 3,7) for the azithromycin group and four days (IQR 3,6; p = 0.49; **S1 File**) for the placebo group.

**Fig 3:**
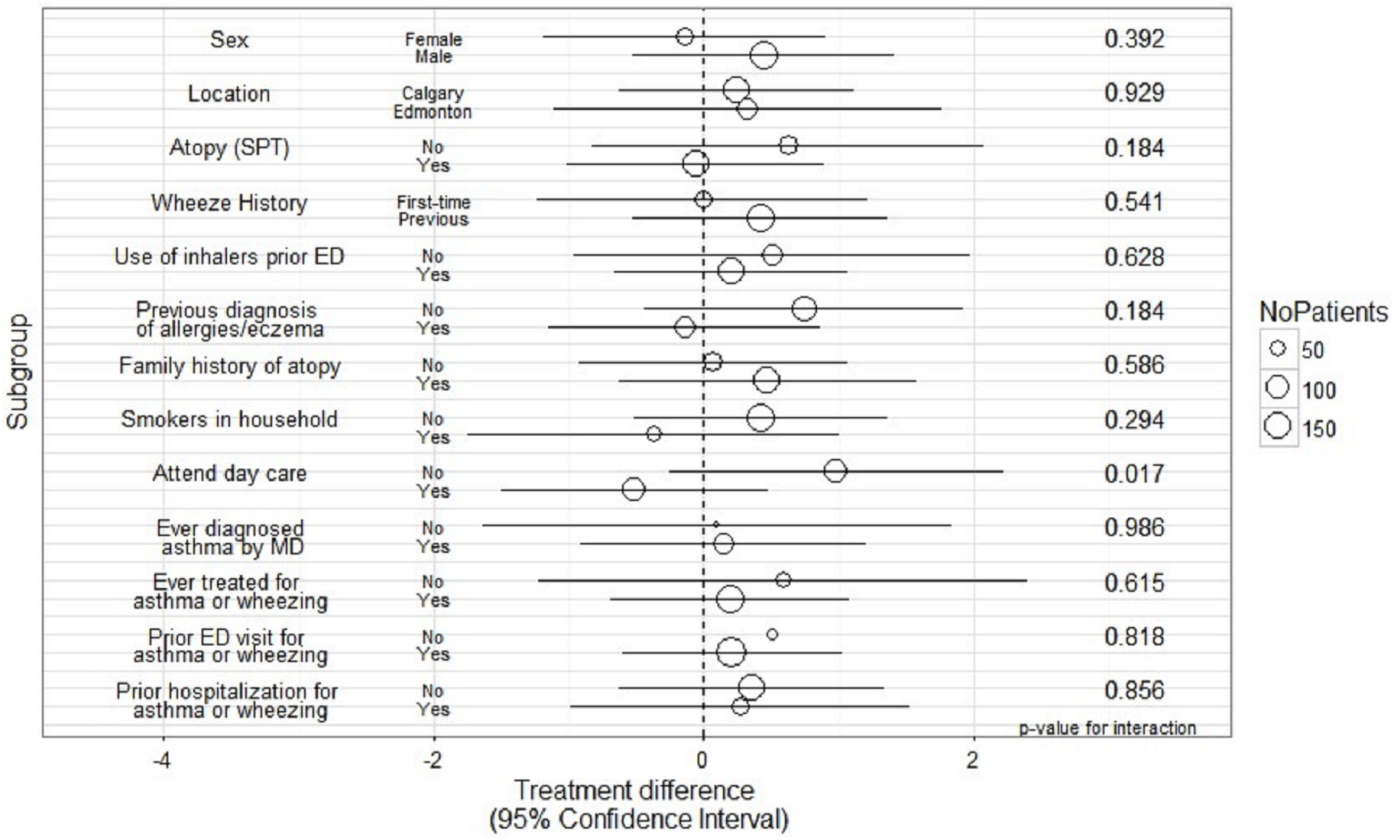
Average change in duration of symptoms (days) in azithromycin versus control treatment arms by subgroup.

For the primary outcome, there was only one significant interaction between treatment effect and day care attendance (p = 0.02). Children who did not attend daycare had a 0.98 day increase in time to resolution of symptoms (95%CI -0.25, 2.22) when treated with placebo versus azithromycin. All other baseline characteristics showed no significant interaction. Specifically, there were no significant interactions between treatment effect and sex, location, atopy, family history, wheezing history, or other environmental characteristics (**Fig 3**).

### Short-acting beta-agonist use (secondary outcome)

The median number of days that a short-acting beta-agonist was used among those who received azithromycin was four and a half days (IQR 2, 7) and five days (IQR 2, 9) among those who received placebo, this difference between drug and placebo arms was not statistically significant (p = 0.22). Similarly, there was no significant difference in the number of days a short-acting beta-agonist was used by prior wheezing status. Among those with first-time wheeze, the median number of days that a short-acting beta-agonist was used among those who received azithromycin was four days (IQR 2, 6) and three days (IQR 0.3, 6.5; p = 0.42) among those who received placebo. Among those with previous wheeze, the median number of days that a short-acting beta-agonist was used among those who received azithromycin was five days (IQR 1.3, 7) and six days (IQR 3, 10; p = 0.10) among those who received placebo.

### Time to disease exacerbation (secondary outcome)

**Fig 4:**
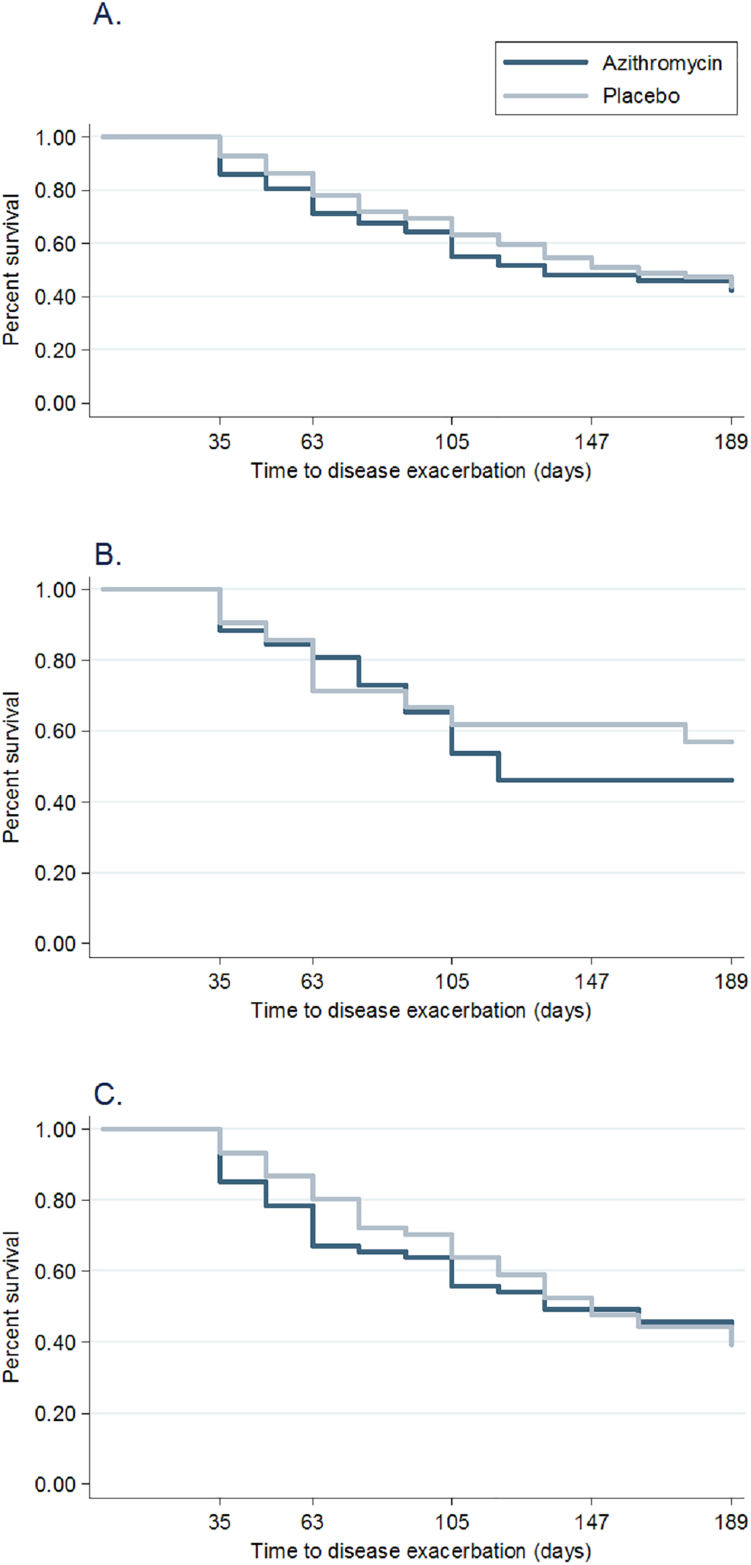
Time to respiratory exacerbation by wheeze category. Kaplan-Meier curve showing time to respiratory disease exacerbation in all randomized participants **(A)**, first-time wheezers **(B)**, and previous wheezers **(C)**. There was no significant difference between pre-school children randomized to azithromycin and placebo.

A total of 96/169 participants (57%) had a disease exacerbation at six-month follow-up. The 189 day exacerbation free rate (**Fig 4**) was not significantly different between treatment groups (hazard ratio 0.91; 95% CI: 0.61 to 1.36; p = 0.65). A sensitivity analysis did not find a significant difference between groups using the log-rank test. Similarly, there was no significant difference in time to disease exacerbation by prior wheezing status (**Fig 4**). Fewer than 50% of those with first-time wheeze (49%, 23/47) presented with an exacerbation (hazard ratio 0.78, 95% CI: 0.34 to 1.80; p = 0.562). Among those with previous wheeze, over 60% (73/122) presented with a disease exacerbation (hazard ratio 0.96, 95% CI: 0.61 to 1.52; p = 0.863). There was no significant interaction between drug and either atopy or family history of atopy for time to disease exacerbation.

### Adverse events

There were a greater number of adverse events reported for placebo (97 events among 139 individuals versus 80 events among 140 individuals for azithromycin) (p = 0.03; **Table 3**). There were no serious or life threatening adverse events and no significant differences by specific symptoms. Two subjects in the azithromycin group discontinued study medication; one due to periorbital erythema and another due to vomiting and diarrhea. The participant with erythema discontinued the study syrup after the first dose but continued regular follow-up in the study. The patient with vomiting and diarrhea was un-blinded by a third-party and the parents were provided with the treatment information. This subject subsequently withdrew from the study.

**Table 3:** Incidence of all reported adverse events.

	Azithromycin (n = 150)	Placebo (n = 150)
Any adverse event	80/140(57.1)	97/139(69.8)
Abdominal pain/discomfort	23/140(16.4)	34/139(24.5)
Nausea/vomit	22/140(15.7)	29/139(20.9)
Headache	8/140(5.7)	14/139(10.1)
Rash	26/140(18.6)	26/139(18.7)
Hematochezia	0/140(0.0)	3/139(2.2)
Diarrhea/loose or watery stools	42/140(30.0)	44/139(31.7)
Jaundice	3/140(2.1)	0/139(0.0)
Hives	10/140(7.1)	16/139(11.5)
Irregular heart rate	10/140(7.1)	4/139(2.9)

Data given as number and percentage (%).

## Discussion

In this multi-center double-blind randomized control trial, we found that azithromycin did not reduce the duration of respiratory symptoms among preschool aged children presenting to the emergency department with wheeze. Azithromycin also did not increase the time to a respiratory exacerbation in the following six months after treatment or reduce short-acting beta agonist medication usage after discharge from the emergency department. There was no significant effect among children with either first-time or prior wheeze. There were no more adverse events for the azithromycin group versus placebo and none of the adverse events were serious or life threatening.

Our results are consistent with a recent Cochrane review that found that antibiotics in general and azithromycin more specifically did not significantly reduce length of hospital stay, duration of oxygen requirement and readmission among infants with bronchiolitis as compared with placebo[17]. Seven total published studies were included in this review; six compared macrolides and three specifically compared azithromycin to placebo. Of the seven studies, six found no difference between antibiotics and placebo. Only one study, with risk of sampling bias due to a small sample of 21 subjects, found that clarithromycin for three weeks significantly reduced hospital admission compared to placebo[20,21].

In contrast to the systematic review and our results, two recently published randomized controlled trials among pre-school children with a history of severe recurrent wheeze showed a significant reduction in the duration of respiratory symptoms and progression to severe lower respiratory tract infection for the acute treatment of respiratory episodes[8,9]. The Stokholm study found that azithromycin reduced the duration of asthma-like episodes in children with recurrent wheezing symptoms from one to three years of age[9]. The Bacharier study concluded that azithromycin for five days, provided early in the course of a respiratory tract infection, prior to presentation to a healthcare provider, prevents the progression to severe lower respiratory tract infection in children with a history of recurrent severe wheezing[8]. Both studies included children at high risk for asthma with recurrent wheeze (at least three previous wheezing episodes during the last 12 month). Our study included children presenting to the emergency department regardless of the severity or chronicity of wheezing symptoms including those children with their first episode with wheeze. Consistent with the Bacharier and Stokholm studies, we reported that azithromycin did not influence the time to subsequent disease exacerbation.

A limitation of this study was our inability to enroll our targeted sample size despite extending the trial completion date by almost three years beyond our anticipated completion date. We experienced low enrolment rates for several reasons: 1) a number of physicians and almost half of families refused to enrol their patients in the study because they were opposed to treating with azithromycin without a clear bacteriological focus 2) a large proportion of children had already been treated with antibiotics in the 30 days prior to presentation (**Fig 1**) 3) few children with the first episode of wheezing presented to the emergency department limiting recruitment. Our sample size is similar to most previous randomized controlled trials evaluating the efficacy of azithromycin for respiratory symptoms[6,7,9,13,22] although the Bacharier study had a higher number of participants included in their analysis[8]. We completed a conditional power analysis using our study’s results. Had we achieved our targeted sample size of 220 participants per group (azithromycin versus placebo) with the same intragroup differences, we would have achieved a 33.7% power to reject the null hypothesis. This analysis suggests that we would be unlikely to reject the null hypothesis even if we had achieved our proposed study sample size.

Macrolide resistance is a world-wide problem with increasing prevalence of resistance in China[23], Japan[24], Europe[25,26] and North America[27,28]. The use of macrolides for the treatment of respiratory symptoms is not recommended without a clear bacteriological background[4]. The Bacharier study reported a slight increase in the number of subjects with azithromycin resistant organisms on patients treated with azithromycin. However, only one site with a total of 81 participants were included in the antimicrobial resistance analysis of the Bacharier study[8].

A recent published Cochrane review of macrolides for chronic asthma recommends that future research should focus on the measurement and report of resistance as an outcome[17]. Future studies could evaluate changes in antibiotic resistance using the nasal swabs collected from all participants (azithromycin and placebo) before the administration of the intervention (enrollment) and 21 days after the first dose.

Our study has several strengths compared to the prior literature. First, enrolment in an emergency department allowed us to include patients evaluated in an acute care setting that attends patients from the general population and not specific sub-groups or high-risk populations such as children with a history of severe lower respiratory tract infections or those diagnosed with recurrent asthma-like symptoms. Second, we completed skin prick testing as an objective assessment of atopic status. Third, we were able to assess both short and long-term impact of azithromycin on respiratory symptoms. Fourth, our pre-planned sub-group analysis allowed us to determine if children with a history of recurrent wheeze responded differently than those children with their first episode of wheeze. Finally, we assessed the practical impact of treating these patients by assessing whether treatment altered children’s daily activities.

## Conclusion

In a multi-center double-blind randomized control trial, azithromycin did not reduce the duration of respiratory symptoms, short-acting beta agonist medication usage after discharge, or time to a respiratory exacerbation among preschool children. We did not find any benefit among first-time or previous wheezers, or among atopic or non-atopic children in a secondary analysis. The number of adverse events in the azithromycin group was not different from placebo and none of the adverse events were serious or life threatening. Based on our data, we do not recommend azithromycin for the regular management of preschool wheezing children presenting to the emergency department.

## Supporting information

S1 File Supporting information. (DOCX)

S2 File CONSORT 2010 checklist. (DOC)

S3 File AZT protocol for publication submission. (PDF)
