# Supplementary material for: Treatment of preschool children presenting to the emergency department with wheeze with azithromycin: A placebo-controlled randomized trial
Source: PLoS One. 2017 Aug 3;12(8):e0182411. doi: 10.1371/journal.pone.0182411 (PMC5542589; doi:10.1371/journal.pone.0182411)
Supplement: S1 File — (DOCX) [file pone.0182411.s001.docx]

**Online supplement: methods**

During enrolment, clinical information, including symptoms of cough, wheeze, rhinitis, and difficulty breathing, was obtained directly from the parents or legal guardian of the child. Emergency department vital signs at admission and discharge, medications prescribed in the emergency department to be used at home, and the final diagnosis and disposition were recorded from the clinical chart (**Appendix Table 2a-j**).

A calendar diary^(14)^ (**Appendix Fig 1**) was used to collect daily respiratory symptoms. Parents were asked to place differently coloured stickers on the calendar corresponding to their child’s respiratory symptoms and to indicate if the study drug was given. The stickers were coded according to the following categories: heart = “no respiratory symptoms”; green star = “mild respiratory symptoms”; yellow star = “respiratory symptoms are worse than usual or more medication than usual was required”; red star = “respiratory symptoms sufficiently severe to require an unscheduled visit to a physician or treatment with oral corticosteroids”; or blue star = “in addition to one of the above and a cold or flu symptoms appear.” Furthermore, star stickers were provided to indicate each day that the study drug was administered. Parents were instructed during enrolment to focus on any respiratory symptoms involving noisy breathing (wheezing or whistling sounds), breathlessness while eating or speaking, shortness of breath after activity or exposure to cold, or persistent troublesome coughing.

The skin prick test (**Appendix Fig 3**) was performed using highly standardized ALK allergens. The same batch of each allergen was used for all subjects. Tree, grass, weed pollen, cat, dog, D. pteronyssinus, D. farinae, cockroach, Alternaria, Cladosporium, Aspergillus, Penicillium, cow’s milk, egg white, soy, wheat and peanut were tested. The wheal and flare (10 minutes for histamine and at 15 minutes for allergens) was outlined in pen and adhesive tape transferred to paper (hard copy). The maximum weal and flare diameters and their mid-point perpendicular responses were measured. The clinical examination (**Appendix Table 1**), based on the Canadian Healthy Infant Longitudinal Development (CHILD) study clinical assessment, was performed to assess for the presence of asthma, allergy or atopic dermatitis^(26)^.

**Online supplement: results**

**Additional demographics:** The most common housing type was house or townhouse with a total of 77.3% participant in the azithromycin group and 80.4% participants in the placebo group (**Appendix Table 2g**). Approximately half of the participant had a pet at home. The most common pet at home was dog, with a total of 34.6% participant in the azithromycin group and 31.3% participants in the placebo group (**Appendix Table 2g**).

A total of 42.7% participant in the azithromycin group and 41.1% participants in the placebo group had a history of allergies or eczema (**Appendix Table 2b**). A total of 47.3% participant in the azithromycin group and 46.4% participants in the placebo group noted an asthma episode at least once (**Appendix Table 2c**). Most participants had their first asthma attack when they were one year old and the last when they were two years old. A total of 42.7% participant in the azithromycin group and 41.1% participants in the placebo group noted eczema at least once (**Appendix Table 2e**). Medication usage characteristics were very well balanced between treatment arms and sub-groups. The most commonly rescue medication used was Ventolin with a total of 86.4% participant in the azithromycin group and 87.5% participants in the placebo group. The most commonly controller medication used was Qvar with a total of 27.3% participant in the azithromycin group and 28.6% participants in the placebo group (**Appendix Table 2h**). The use of rescue medication was very well balanced between first-time and previous wheezers by treatment arm sub-groups. However, previous wheezers presented higher use of rescue medication before activity (**Appendix Table 2j**). The most common previous history was hayfever in both parents (**Appendix Table 2f**).

**Cough and phlegm symptoms:** Cough and phlegm history were very well balanced between treatment arms and sub-groups. A total of 48.2% participant in the azithromycin group and 52.7% participants in the placebo group had coughed at night without a cold at least once (**Appendix Table 2e**). Cough and phlegm characteristics were also well balanced between first time wheezers and previous wheezers by treatment arm sub-groups (**Appendix Table 2j**).

**First-time wheezer versus prior wheezer:** Participants were mostly males in the first-time wheezers and previous wheezers subgroups: 74.2% males first-time wheezers in the azithromycin group and 69.7% males first-time wheezers in the placebo group; 68.4% males previous wheezers in the azithromycin group and 76.0% males previous wheezers in the placebo group. The mean age in months among first-time wheezers in the azithromycin group was 31.0 (SD: 12.7) and 26.5 (13.1) in the placebo group; 36.2 (13.8) among previous wheezers in the axithromycin group and 32.2 (13.9) in the placebo group (**Appendix Table 2j**). A total of 48.4% first-time wheezers in the azithromycin group and 66.7% first-time wheezers in the placebo group had a previous episode of shortness of breath; while 78.5% previous wheezers in the azithromycin group and 77.2% previous wheezers in the placebo group had previous episode of shortness of breath. The frequency of shortness of breath was noted as rarely in first-time wheezers and rarely/repeatedly in previous wheezers (**Appendix Table 2j**).

A total of 22.6% first-time wheezers in the azithromycin group and 24.2% first-time wheezers in the placebo group had a previous asthma episode; while 57.0% previous wheezers in the azithromycin group and 55.7% previous wheezers in the placebo group had previous asthma episode. The age of first and last asthma attack were very well balanced between treatment arms. The age of first and last asthma attacks in first-time wheezers is spread among 1 to 5 years old. Previous wheezers had their first asthma attack when they were one year old and the last asthma attack when they were two years old (**Appendix Table 2j**). Only 20% (n=13) first-time wheezers and 81% (n=128) previous wheezers had used an inhaler previous to the emergency department (**Appendix Table 2j**).

**Online supplement: Funding**

Grant support was received for the intervention from the Alberta Lung Association (ALA). The funders had no role in study design, data collection and analysis, decision to publish, or preparation of the manuscript.

**Appendix Tables and Figures**

Appendix Table 1: Study Design

Appendix Table 2a - j: Expanded demographics and discharge medication

Appendix Table 3: Mean time to resolution of symptoms (primary outcome) by treatment group

Appendix Figure 1: Daily Diary

Appendix Figure 2: Distribution of time to resolution of symptoms among treatment groups.

Appendix Figure 3: Skin prick test

**Appendix Table 1: Study Design**

| **Data Collection Instrument** | **Enrolment and Randomization** | **ED Discharge Vitals** | **Follow-up Day1** | **Follow-up Day3** | **Follow-up Day5** | **Follow-up Day14** |
| --- | --- | --- | --- | --- | --- | --- |
| Emergency Enrolment | x |  |  |  |  |  |
| Emergency Discharge |  | x |  |  |  |  |
| Follow-up |  |  | x | x | x | x |
| In-Clinic Visit |  |  |  |  |  |  |
| - Daily Diary |  |  |  |  |  |  |
| - Clinical Assessment |  |  |  |  |  |  |
| - Baseline Questionnaire |  |  |  |  |  |  |
| - Skin Prick Test |  |  |  |  |  |  |
| Monitoring Adverse Events | x | x | x | x | x | x |

| **Data Collection Instrument** | **In-Clinic Visit Day21** | **Follow-up Day 35** | **Follow-up Day63** | **Follow-up Day105** | **Follow-up Day 147** | **Follow-up Day189** |
| --- | --- | --- | --- | --- | --- | --- |
| Emergency Enrolment |  |  |  |  |  |  |
| Emergency Discharge |  |  |  |  |  |  |
| Follow-up | x | x | x | x | x | x |
| In-Clinic Visit |  |  |  |  |  |  |
| - Daily Diary | x |  |  |  |  |  |
| - Clinical Assessment | x |  |  |  |  |  |
| - Baseline Questionnaire | x |  |  |  |  |  |
| - Skin Prick Test | x |  |  |  |  |  |
| Monitoring Adverse Events | x | x |  |  |  |  |

**Appendix Table 2a: Demographic Characteristics – All Randomized Participants**

| **Participants/Characteristics** | **Not included for Primary Outcome**  **(N=78)** | **Not included**  **Azithromycin**  **(N=40)** | **Not included**  **Placebo**  **(N=38)** | **Included for Primary Outcome**  **(N=222)** | **Included**  **Azithromycin (N=110)** | **Included**  **Placebo**  **(N=112)** |
| --- | --- | --- | --- | --- | --- | --- |
| Male (n (%)) | 55 (70.5) | 30 (75.0) | 25 (65.8) | 159 (72.0) | 77 (70.0) | 83 (74.1) |
| Age in months – mean (SD) | 31.5/75 (16.0) | 31.2/37 (17.6) | 31.8/38 (14.5) | 32.6 (13.9) | 34.8 (13.6) | 30.5 (13.9) |
| Cough at triage (n (%)) | 72 (92.3) | 39 (97.5) | 33 (86.8) | 217 (97.7) | 109 (99.1) | 108 (96.4) |
| Inhalers used prior ED (n (%)) | 42 (53.9) | 22 (55.0) | 20 (52.6) | 141 (63.5) | 73 (66.4) | 68 (61.7) |
| Previous Wheezing History (n(%)) | 58 (74.4) | 29 (72.5) | 29 (76.3) | 158 (71.2) | 79 (71.8) | 78 (70.5) |

Data given as number or mean, percentage (%) or standard deviation (SD) as appropriate.

**Appendix Table 2b: Expanded Demographics**

| **Demographics** | **Azithromycin**  **(N=110)** | **Placebo**  **(N=112)** |
| --- | --- | --- |
| Male (n (%)) | 77 (70.0) | 83 (74.1) |
| **Age in months – m(SD)** | 34.8 (13.6) | 30.5 (13.9) |
| Symptoms: triage |  |  |
| Cough | 109 (99.1) | 108 (96.4) |
| Runny nose | 90 (81.8) | 97 (86.6) |
| Wheezy | 100 (100.0) | 111 (99.1) |
| Difficulty breathing | 106 (96.4) | 104 (92.9) |
| **Participants/Previous Medical History** |  |  |
| Dx allergies/eczema | 47 (42.7) | 46 (41.1) |
| Family Hx of atopy | 69 (62.7) | 66 (58.9) |
| Previous significant illness | 33 (30.0) | 20 (17.9) |
| Required intubation in the past | 6 (5.5) | 5 (4.5) |
| Smokers in household | 26 (23.6) | 20 (17.9) |
| Regular exposure to smoke outside household | 8 (7.3) | 8 (7.1) |
| Attend day care | 49 (44.6) | 53 (47.3) |

Data given as number and percentage (%).

**Appendix Table 2c: Expanded Demographics – Asthma history**

| **Asthma history** | **Azithromycin**  **(N=110)** | **Placebo**  **(N=112)** |
| --- | --- | --- |
| Ever asthma | 52 (47.3) | 52 (46.4) |
| Ever diagnosed asthma by MD | 45 (40.9) | 39 (34.8) |
| Ever had an asthma attack | 50 (45.5) | 49 (43.8) |
| Ever had any treatment for asthma or wheezing | 74 (67.3) | 84 (75.0) |
| Ever had any treatment for asthma or wheezing in the last 12 months | 73 (66.4) | 79 (70.5) |
| ED visit for asthma or wheezing | 91 (82.7) | 97 (86.6) |
| Hospitalized for asthma or wheezing | 36 (32.7) | 27 (24.1) |
| Use of inhaler prior ED | 73 (66.4) | 68 (61.7) |
| Number of inhalers used prior ED |  |  |
| 1 | 32 (29.1) | 28 (25.0) |
| 2 | 37 (33.6) | 36 (32.1) |
| 3 | 4 (3.6) | 4 (3.6) |
| Inhalers used prior ED |  |  |
| Short-acting beta agonist | 69 (62.7) | 66 (58.9) |
| Inhaled Corticosteroids | 39 (35.5) | 41 (36.6) |

Data given as number and percentage (%).

**Appendix Table 2d: Expanded Demographics – Wheeze and SOB history**

| **Wheeze and SOB history** | **Azithromycin**  **(N=110)** | **Placebo**  **(N=112)** |
| --- | --- | --- |
| Wheeze and chest tightness |  |  |
| Ever wheeze before* | 97 (88.2) | 98 (87.5) |
| Wheeze without a cold | 52 (47.3) | 43 (38.4) |
| Breathless when wheezing ever | 66 (60.0) | 69 (61.6) |
| Wheeze during the last year | 89 (80.9) | 89 (79.5) |
| Woken up w/tightness of chest  during last 12 months | 43 (39.1) | 55 (49.1) |
| Shortness of breath |  |  |
| Shortness of breath ever | 77 (70.0) | 83 (74.1) |
| SOB during the day at rest | 34 (30.9) | 31 (27.7) |
| SOB during the day at rest during the  last 12 months | 33 (30.0) | 29 (25.9) |
| SOB during moderate physical activity  during the last 12 months | 27 (24.6) | 21 (18.8) |
| SOB following strenuous activity  during the last 12 months | 41 (37.3) | 36 (32.1) |
| Woken up at night by an attack of SOB  during the last 12 months | 39 (35.5) | 45 (40.2) |
| Frequency of SOB |  |  |
| Rarely | 40 (36.4) | 50 (44.6) |
| Repeatedly | 34 (30.9) | 29 (25.9) |
| Continuously | 3 (2.7) | 4 (3.6) |

Data given as number and percentage (%).

**Appendix Table 2e: Expanded Demographics – Cough, phlegm, and other symptoms history**

| **Cough, phlegm, and other symptoms history** | **Azithromycin**  **(N=110)** | **Placebo**  **(N=112)** |
| --- | --- | --- |
| Ever coughed at night without a cold | 53 (48.2) | 59 (52.7) |
| Woken up coughing without a cold (last 12 months) | 43 (39.1) | 47 (42.0) |
| Usually cough on getting up (last 12 months) | 29 (26.4) | 28 (25.0) |
| Coughing on getting up most mornings for at least 3 months in a row each year | 11 (10.0) | 7 (6.3) |
| Usually phlegm on getting up(last 12 months) | 9 (8.2) | 8 (7.1) |
| Phlegm on getting up most mornings for at least 3 months in a row each year | 6 (5.5) | 2 (1.8) |
| Hayfever symptoms ever | 30 (27.3) | 38 (34.8) |
| Hayfever symptoms during the last  12 months | 23 (20.9) | 37 (33.0) |
| Eczema ever | 53 (48.2) | 49 (43.8) |
| Eczema during the last 12 months | 42 (38.2) | 43 (38.4) |
| Urticaria ever | 38 (34.6) | 43 (38.4) |
| Urticaria during the last 12 months | 27 (24.6) | 38 (33.9) |
| Other allergies ever | 28 (25.5) | 32 (28.6) |
| Other allergies during the last 12  months | 19 (17.3) | 17 (15.2) |

Data given as number and percentage (%).

**Appendix Table 2f: Expanded Demographics – Family history**

| **Family atopy history** | **Azithromycin**  **(N=110)** | **Placebo**  **(N=112)** |
| --- | --- | --- |
| **Participants/Father’s history** |  |  |
| Prev Hx of asthma | 29 (26.4) | 28 (25.0) |
| Ever had hayfever symptoms | 35 (31.8) | 39 (34.8) |
| Ever had eczema | 22 (20.0) | 24 (21.4) |
| Ever had other allergies | 37 (33.6) | 36 (32.1) |
| **Participants/Mother’s history** |  |  |
| Prev Hx of asthma | 30 (27.3) | 33 (29.5) |
| Ever had hayfever symptoms | 29 (26.4) | 48 (42.9) |
| Ever had eczema | 30 (27.3) | 37 (33.0) |
| Ever had other allergies | 51 (46.4) | 51 (45.5) |

Data given as number and percentage (%).

**Appendix Table 2g: Expanded Demographics – Environmental history**

| **Participants/House type** | **Azithromycin**  **(N=110)** | **Placebo**  **(N=112)** |
| --- | --- | --- |
| Apartment building | 13 (11.8) | 9 (8.0) |
| House or townhouse | 85 (77.3) | 90 (80.4) |
| Duplex | 5 (4.6) | 8 (7.1) |
| Farm with animals | 1 (0.9) | 2 (1.8) |
| Pets at home | 58 (52.7) | 53 (47.3) |
| Dogs | 38 (34.6) | 35 (31.3) |
| Cats | 23 (20.9) | 19 (17.0) |
| Others | 12 (10.9) | 17 (15.2) |

Data given as number and percentage (%).

**Appendix Table 2h: Expanded Rescue and Controller Medication**

|  | **Azithromycin**  **(N=110)** | **Placebo**  **(N=112)** |
| --- | --- | --- |
| **Rescue medication:** |  |  |
| Short-acting beta2-agonist (SABA) |  |  |
| Ventolin | 95 (86.4) | 98 (87.5) |
| Airomir | 1 (0.9) | 0 (0.0) |
| Salbutamol Nebule | 0 (0.0) | 3 (2.7) |
| Use of rescue medication  before activity | 11 (10.0) | 15 (13.4) |
| **Controller medication:** |  |  |
| Inhaled Corticosteroids |  |  |
| Flovent | 28 (25.5) | 28 (25.0) |
| Pulmicort | 1 (0.9) | 1 (0.9) |
| Becloforte | 1 (0.9) | 0 (0.0) |
| Qvar | 30 (27.3) | 32 (28.6) |
| Advair | 2 (1.8) | 0 (0.0) |
| Alvesco | 9 (8.2) | 10 (8.9) |
| Anticholinergic |  |  |
| Atrovent | 2 (1.8) | 3 (2.7) |
| Leukotriene receptor antagonist (LTRA) |  |  |
| Singulair | 6(5.5) | 5(4.5) |

Data given as number and percentage (%).

**Appendix Table 2i: Emergency department and discharge medication**

| **Emergency department medication** | **Azithromycin**  **(N=110)** | **Placebo**  **(N=112)** |
| --- | --- | --- |
| Salbutamol | 106 (96.4) | 107 (95.5) |
| Atrovent | 92 (83.6) | 82 (73.2) |
| Other bronchodilators | 8 (7.3) | 8 (7.1) |
| Steroids | 94 (85.5) | 90 (80.4) |
| Epinephrine | 0 (0.0) | 3 (2.7) |
| **Discharge medication** |  |  |
| SABA | 87 (79.1) | 82 (73.2) |
| Oral corticosteroids | 65 (59.1) | 70 (62.5) |
| Inhaled corticosteroids | 63 (57.3) | 57 (50.9) |

Data given as number and percentage (%).

**Appendix Table 2j: Baseline Demographics by first versus previous wheezer**

|  | **Azithromycin First Time Wheezy (N=31)**  **n(%)** | **Placebo**  **First Time Wheezy (N=33)**  **n(%)** | **Azithromycin**  **Previous Wheezy (N=79)**  **n(%)** | **Placebo**  **Previous Wheezy (N=79)**  **n(%)** |
| --- | --- | --- | --- | --- |
| Male | 23 (74.2) | 23 (69.7) | 54 (68.4) | 60 (76.0) |
| Age in months | 31.0 (12.7) | 26.5 (13.1) | 36.2 (13.8) | 32.2 (13.9) |
| Ever shortness of breath | 15 (48.4) | 22 (66.7) | 62 (78.5) | 61 (77.2) |
| Ever asthma attack | 7 (22.6) | 8 (24.2) | 45 (57.0) | 44 (55.7) |
| Ever diagnosed asthma | 5 (16.1) | 4 (12.1) | 40 (50.6) | 35 (44.3) |
| Use of inhaler prior ED | 7 (22.6) | 6 (18.2) | 66 (83.5) | 62 (78.5) |
| Dx allergies/eczema | 11 (35.5) | 15 (45.5) | 36 (45.6) | 31 (39.2) |
| Ever coughed at night without a cold | 10 (32.5) | 17 (51.5) | 43 (54.4) | 42 (53.2) |
| Usually phlegm on getting up (last 12 months) | 2 (6.5) | 2 (6.1) | 7 (8.9) | 6 (7.6) |
| Family Hx of atopy | 18 (58.1) | 19 (57.6) | 51 (64.6) | 47 (59.5) |
| Smokers in household | 7 (22.6) | 4 (12.1) | 19 (24.1) | 16 (20.3) |
| Attend day care | 12 (38.7) | 15 (45.5) | 37 (46.8) | 38 (48.1) |
| House or townhouse | 24 (77.4) | 27 (81.8) | 61 (77.2) | 63 (79.8) |
| Pets at home | 17 (54.8) | 19 (57.6) | 41 (51.9) | 34 (43.0) |

Data given as number and percentage (%).

**Appendix Table 3: Median time to resolution of symptoms (primary outcome) by treatments**

|  | | **Median time (days) (95% CI)** | |  | |
| --- | --- | --- | --- | --- | --- |
|  | **Azithromycin** | | **Placebo** | | **p-value** |
| Total Sample (n=222) | 4 (3 to 6) | | 4 (3 to 6) | | 0.2827 |
| First time wheeze (n=64) | 4 (3 to 6) | | 4 (3 to 5) | | 0.3972 |
| Prior wheezer (n=158) | 4 (3 to 7) | | 4 (3 to 6) | | 0.4867 |
| Non-atopic (n=70) | 5 (3 to 7) | | 4.5 (3 to 7) | | 0.9427 |
| Atopic (n=138) | 4 (3 to 6) | | 4 (3 to 5) | | 0.0470 |

**Appendix Figure 1: Daily Diary**


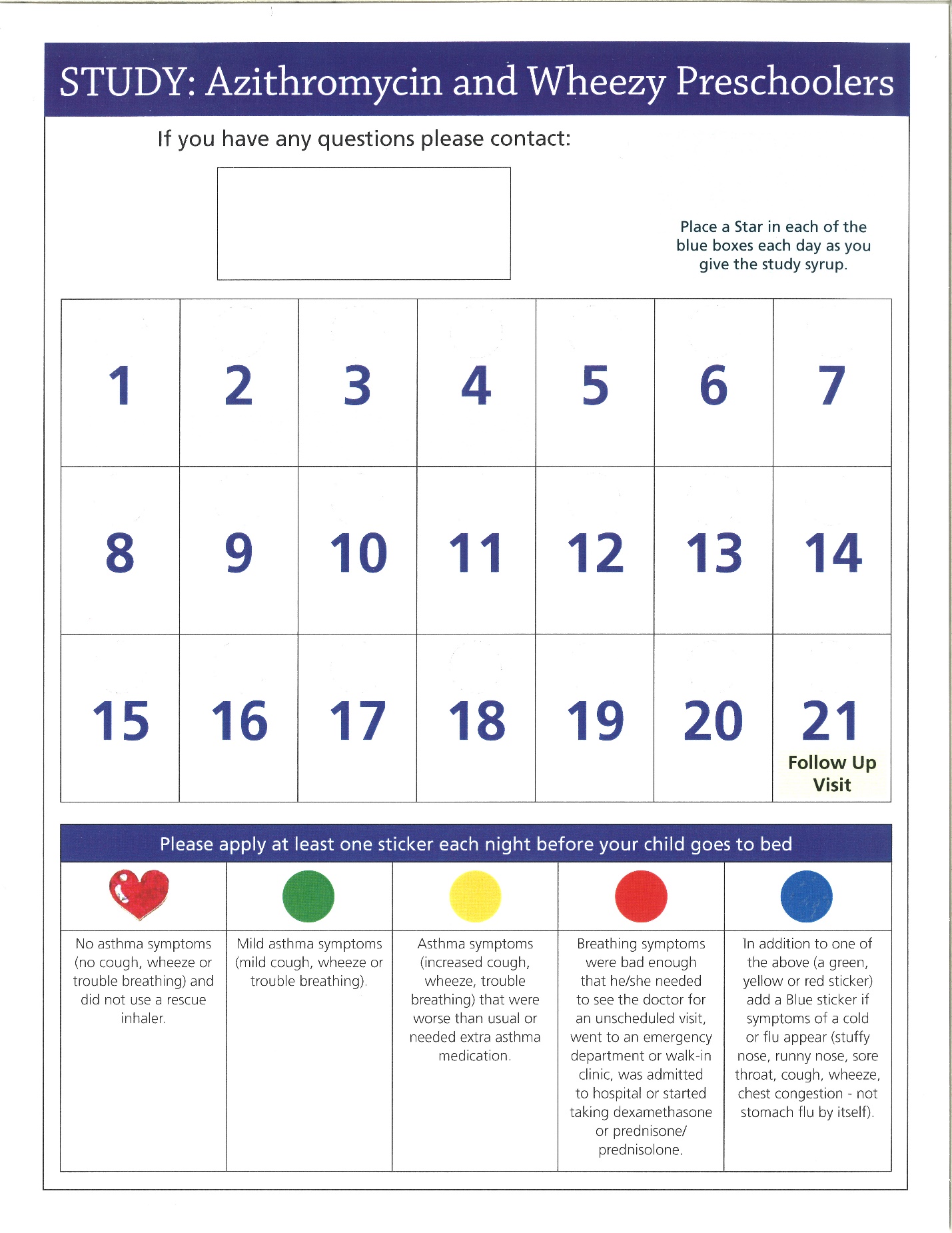


**Appendix Figure 2: Distribution of time to resolution of symptoms among treatment groups.**

**Appendix Figure 3: Skin prick test**
