# Supplementary material for: Treatment of preschool children presenting to the emergency department with wheeze with azithromycin: A placebo-controlled randomized trial
Source: PLoS One. 2017 Aug 3;12(8):e0182411. doi: 10.1371/journal.pone.0182411 (PMC5542589; doi:10.1371/journal.pone.0182411)
Supplement: S3 File — (PDF) [file pone.0182411.s003.pdf]

Title of the proposal: **A double-blind, randomized control trial of azithromycin for the acute management of wheezy pre-school children**

1. THE PROBLEM

One in three children wheeze prior to their third birthday; almost 50% of children wheeze by 6 years of age [1, 2]. Between 1996 and 2005, while Ontario emergency department (ED) visits for asthma decreased, the ED visits for children 0 to 4 years did not change (14.6 visits / 100 asthma individuals in 1996 versus 14.4 visits / 100 asthma individuals in 2005; *Figure 1*). Children aged 0-4 years also had the highest asthma admission rates in Ontario. Preschool children who wheeze use 0.15% of the total healthcare budget in the UK [3]. **Preschool wheeze represents significant morbidity for the individual and a significant burden to society.**

Preschool children who wheeze represent a number of heterogeneous populations and disorders including: 1) the majority of children who will only wheeze with viral infections and only in childhood (episodic wheeze) 2) children who will wheeze due to multiple triggers (a potential pre-cursor phenotype of asthma) and 3) children who wheeze secondary to upper airway infections such as rhino-sinusitis. These multiple, overlapping etiologies are difficult to distinguish and make the acute and chronic management of children with preschool wheeze challenging [4]. **Many current drug therapies target inflammation as a potential cause of preschool wheeze but do not address atypical bacterial infection as a potential contributing factor to the observed morbidity.**

2. OVERALL AIM

We hypothesize that the addition of azithromycin, a macrolide antibioitic with anti-inflammatory [5, 6] and anti-bacterial [7] properties, to routine therapy of wheezy pre-school children – regardless of aetiology - will resolve respiratory symptoms more rapidly, reduce the need for rescue medications in the subsequent three weeks, and protect against symptom reoccurrence longer than the addition of placebo.

To test this hypothesis, **we propose a double-blind, randomized control trial (RCT) of 5 days of azithromycin versus placebo in preschool children (between 12 and 60 months of age) presenting to the pediatric ED with wheezing symptoms**.

3. BACKGROUND LITERATURE

Challenges in managing preschool wheeze: While many physicians label preschool children with recurrent wheeze as having asthma, some experts now distinguish two patterns of disease: episodic and multiple-trigger wheeze [4, 8]. Episodic wheeze is defined as wheeze in discrete episodes, with the child being well between episodes. Although not unique to the preschool age group [9, 10], this phenotype appears to be most common in preschool children [1, 11, 12]. Multi-trigger wheeze is defined as those children who have persistent respiratory symptoms in between acute wheezing episodes. These children's symptoms are triggered by multiple triggers such as allergens, exercise, and cold air. Unfortunately, a viral respiratory tract infection is the most common trigger factor for wheeze in preschool children regardless of the episodic or multiple trigger classification [13]. This makes it difficult to distinguish children who wheeze due to multiple-triggers from those children with episodic wheeze.

In addition to episodic and multiple-trigger wheeze, some experts believe a child's response to treatment with their first episode of wheeze (bronchiolitis)[14] is different than if

they had a prior episode of wheeze. Others believe that a child's response to treatment with their first wheeze is likely to be heterogeneous, and more dependent on whether they have an intrinsic predisposition to have episodic or multiple-trigger wheeze.[4] Current evidence is insufficient to be determine if either or both views are true.

In addition to episodic, multiple-trigger, and first-time wheeze, co-morbid conditions such as rhino-sinusitus may contribute to wheeze symptoms [8]. The presence of chronic rhinitis and sinusitis is frequently diagnosed in children with asthma [15]. Treatment of the upper airway conditions in patients with asthma improves the function of the lower airway [16-18], although the mechanism by which lower airway function improves remains theoretical. Potential mechanisms by which rhino-sinusitis may influence preschool wheeze include a combination of systemic inflammatory signals, breathing mechanics, or bacterial contaminants from the upper airway entering the lung [15].

Aetiology and pathophysiology: A recent European Respiratory Society (ERS) taskforce concluded that the pathogenesis of pre-school children who wheeze has been inadequately studied, and that many preschool children who wheeze may not have the classic inflammatory changes characteristic of older children and adults with asthma [19]. Preschool wheeze is usually associated with clinical evidence of a viral respiratory tract infection. The most common infectious agents include rhinovirus, respiratory syncytial virus (RSV), coronavirus, human metapneumovirus, parainfluenza and adenovirus [13]. It is unclear what role bronchospasm and bronchial smooth muscle hyperreactivity play versus airway inflammation, edema and accumulation of debris in the development of clinical signs of wheeze [20, 21]. Few studies have investigated the presence of airway inflammation in preschool wheezy children. Elevated exhaled nitric oxide fractions (F_{eNO}), an indirect marker of airway inflammation, have been found in wheezing infants, especially when they are atopic [22, 23], and F_{eNO} levels normalise during treatment with inhaled corticosteroid (ICS) [23] or leukotriene receptor antagonists (LTRA) such as montelukast [24, 25].

Pharmacological therapy: The current management of children who wheeze includes supplemental oxygen, hydration and nutrition [4]. Inhaled short acting β_2-agonists (SABA) are the drugs of choice for acute symptoms of wheeze. RCT of SABA in infants and preschool children have demonstrated significant bronchodilator effects [26-29] and protective effects against broncho-constrictor agents [30, 31] . While SABA are effective acutely, they require frequent administration and do not influence the underlying disease pathogenesis.

Systematic reviews are available that evaluate the utility of ICS, LTRA or long acting beta-agonists (LABA) to treat children with asthma. Preschool wheeze may not represent the same pathophysiology as asthma and the role for these drugs to treat preschool wheeze may be different. Several studies have examined the role of ICS and LTRA to modify the underlying disease pathogenesis (presumed inflammation and airway hyperreactivity) of wheezing preschool children. There have been no trials of LABA [30, 32] or macrolides for the acute treatment of preschool wheeze.

<u>*ICS and oral corticosteroids:*</u> The benefits of ICS may depend on whether children wheeze episodically or whether they wheeze in response to multiple triggers. A recent meta-analysis demonstrated improvement in wheezing symptoms and time to next wheeze/asthma exacerbation in preschool children with recurrent wheeze treated for 4 to 6 weeks with ICS [33]. The benefits of ICS for episodic wheeze are more controversial [34]. Systematic reviews have concluded that episodic high-dose ICS results in a 50% reduction in the requirement for oral steroids but without an effect on hospitalisation rates or duration of symptoms. The most

recent study showed that intermittent treatment with high dose ICS at the start of a respiratory tract infection (RTI) reduced the severity and duration of symptoms but at a cost of reduced height and weight gain [35]. Maintenance treatment with low-dose ICS does not appear to reduce the number or the severity of wheezing episodes in episodic (viral) wheeze [34, 36 37]. Similar results were found with the use of oral prednisolone in preschool children with episodic wheeze who present to a hospital [38]. A systematic review of two studies found no evidence that parent-initiated oral corticosteroids are associated with a benefit in terms of hospital admissions, unscheduled medical reviews, symptoms scores, bronchodilator use, parent and patient impressions, physician assessment, or days lost from work or school [39]. As it is difficult to distinguish children with episodic versus multiple-trigger wheeze, treating all wheezing preschool children with corticosteroids would treat those with episodic wheeze (the majority) with a drug that may not be effective and can have adverse effects.
Nasal corticosteroids: A randomised controlled trial of nasal corticosteroids in preschool children with recurrent wheeze failed to demonstrate any benefit [40].
LTRA: Daily use of Montelukast over a 1-yr period reduced the rate of wheezing episodes among 2 to 5 year old children by 32% compared to placebo (number needed to treat 12) [41]. montelukast started when patients developed signs of a RTI also showed a 30% reduction in unscheduled health visits (number needed to treat 11), but no effect on hospitalisations, duration of episode, and SABA and prednisolone use [42]. We have shown that Montelukast taken daily for 45 days over the period of the September epidemic reduces unscheduled physician visits by 78% compared to placebo (number needed to treat 10) [43]. While LTRA have been shown to be effective in preschool children clinically and in reducing airway inflammation, LTRA lack anti-bacterial properties which may be important in the management of wheezing preschool children.

Macrolide antibiotics in the management of preschool wheeze: Macrolide antibiotics (e.g. clarithromycin, azithromycin) demonstrate anti-inflammatory effects in animal and human studies [44]. Macrolides may reduce airway inflammation by reducing airway mucus secretion [45] and decreasing airway neutrophil accumulation through a reduction in both pro-inflammatory cytokines and adhesion molecule production [46-48]. A Cochrane review of macrolide therapy in chronic asthma management showed significant differences in eosinophilic inflammation and symptoms [49]. In a randomized control trial of adults with severe, refractory asthma, clarithromycin significantly reduced airway concentrations of interleukin (IL)-8, metalloproteinase (MMP)-9, neutrophil elastase, neutrophil numbers, and improved quality-of-life scores compared with those adult asthmatics on placebo [50]. In a mouse model of allergic asthma, azithromycin attenuated ovalbumin-dependent airway inflammation [51]. While we could not identify any RCT of macrolide antibiotics in preschool wheeze with clinically relevant outcomes, macrolide antibiotics have been tested in several other pediatric respiratory diseases.
Chronic rhino-sinusitis: Macrolide treatment of chronic rhino-sinusitis has shown significant improvement in a variety of outcomes at 3 and 12 month follow [52-56].
RSV Bronchiolitis: A RCT of clarithromycin for 3 weeks in 21 infants with RSV bronchiolitis showed a significant reduction in length of hospital stay, duration of supplemental oxygen, need for SABA, and readmission to the hospital within 6 months after discharge [57]. Infants treated with clarithromycin also showed significant decreases in plasma IL-4, IL-8 and eotaxin levels [58]. Inclusion of children who present with first-time wheeze (i.e. bronchiolitis) will allow us to expand on the preliminary results of this study.

Acute asthma exacerbations: A RCT of clarithromycin in 4 – 17 year old children (n=43) with an acute exacerbation of either recurrent wheezing or asthma showed significantly lower nasopharyngeal concentrations of TNF-α, IL-1β, and IL-10 up to 8 weeks after treatment [59]. Unfortunately all children were treated with systemic steroids and symptom outcomes were not reported. Adults with an asthma exacerbation treated for 10 days with telithromycin showed a significant reduction in asthma symptoms and improvement in pulmonary function at the end of treatment. While symptom free days continued to improve, differences in pulmonary function were not sustained at 6-week follow-up [60].

The efficacy of macrolide antibiotics on acute wheezing symptoms may depend on the presence of atypical bacterial infection (*Mycoplasma pneumoniae* or *Chlamydophila pneumoniae)*. Atypical organisms have been associated with exacerbations of asthma in both children and adults [61-63]. A case control study of children (age 2 to 14 years) with an acute wheezing episode (n = 71) showed that *M. pneumoniae* and *C. pneumoniae* infections were detected significantly more often in children with wheezing symptoms than in healthy controls. During a 3-month follow-up period, those children with *M. pneumoniae* or *C. pneumoniae* showed a significantly greater recurrence of wheeze than those without atypical bacteria present [64]. *C pneumoniae* or *M pneumoniae* was found in 53% of patients in the pediatric clarithromycin and asthma trial [59]. In the adult, acute asthma exacerbation RCT, although 61% of the study population that used telithromycin had evidence of *C. pneumoniae* or *M. pneumoniae*, bacteriological status was not related to telithromycin response [60]. However, many respiratory pathogens are not detected or are easily overlooked by routine clinical microbiology [65, 66]. These studies suggest that macrolide antibiotics may have an important role in the management of wheeze in children.

Potential adverse events associated with macrolide use: Macrolide antibiotics have been associated with significant effects on cardiac conduction [67], increased antimicrobial resistance [68], and an increase in liver enzymes among those who have abnormal liver enzyme levels prior to treatment [60]. Statin-induced myopathy has been reported with concurrent clarithromycin therapy [69]

Macrolide antibiotics possess pro-arrhythmogenic effects [70, 71] leading to prolong cardiac repolarisation [72-74] and potentially fatal ventricular tachycardia [75]. The risk is increased five-fold if patients are concurrently taking cytochrome P_{450}3A inhibitors [70.] Newer macrolide antibiotics such as azithromycin, may be associated with a reduced risk of cardiac arrhythmias [76, 77 78]. The risk related to a short course of macrolide treatment for an acute infection in a young person might be different to the risk related to prolonged use in an older patient who is more likely to both have structural cardiac disease and be taking other commonly used drugs that prolong the QT interval [79, 80].

Macrolide use is the most important driver of macrolide resistance.[81, 82] Macrolide resistant *Streptococcus pneumoniae* is increasing in Europe [83, 84 85]. Azithromycin selects for a greater number of resistant organisms while clarithromycin selects a higher-resistance-coding gene mutation. The potential for resistance development may be greater with azithromycin compared to clarithromycin [68].

Which macrolide to use: Comparative studies of the relative clinical efficacy of different macrolide therapies have not been completed. Both azithromycin and clarithromycin are associated with increased microbial resistance. Differences in the anti-inflammatory properties are not informative as little is known about the immuno-patho-physiology of pre-school wheeze. Azithromycin has been used in the management of rhino-

sinusitis [56, 86] and may be associated with less cardiac conduction effects. It is for the latter two reasons that we chose azithromycin for this trial.

4. STUDY OBJECTIVE

To determine if treatment of pre-school children with a history of wheeze who present to an ED with an acute wheezing episode with azithromycin for 5 days will resolve their symptoms more quickly, will use less SABA, and allow these children to remain symptom free for a longer period of time.

5. HYPOTHESES

Primary Hypothesis: Treatment of pre-school children with an acute episode of wheezing symptoms with 5 days of azithromycin will resolve their symptoms sooner.

Secondary Hypotheses: Treatment of pre-school children with acute wheezing symptoms with 5 days of azithromycin will: a) cause these children to use less rescue β2 agonists than those treated with placebo; and b) allow these children to remain free of subsequent wheezy episodes longer than those treated with placebo.

6. RESEARCH DESIGN

Study Overview and Setting: We propose a two arm, double blind, randomized placebo controlled trial conducted in two urban pediatric emergency departments (Alberta Children's Hospital (ACH), Calgary and Stollery Children's Hospital (SCH), Edmonton). *(Appendix A – Study Design Schema)*

***Patient recruitment*:** Starting January, 2010, trained research assistants will be on-call 16 hours per day, seven days per week (*Appendix B – Emergency Medicine Research Associates Program*). The triage nurse will notify on-call research assistants about children 12-60 months of age who present with wheeze during the study period. Written, informed consent will be obtained as follows: 1) a video of a primary investigator explaining the study will be played for the parent (or legal guardian) by the assistants, 2) a primary investigator or senior research coordinator will be available by telephone to answer any questions the parent/legal guardian might have about the study 3) the research assistant will ask the parent/legal guardian who have verbally agreed to participate to sign the written consent form (*Appendix C – Consent Form*). A more lay description of the study including rationale, objectives and the study commitment will also be distributed as a brochure.

We propose to implement a lottery remuneration process within the ED. For each individual screened, the research assistant, admitting clerk, the nurse facilitating the enrollment visit can complete a ballot for entry. The ballot will contain the patient's bed number, time and date of the patient encounter. RA will check the handed ballot. The entry for RA will be confirmed through check box. The lottery draw will be completed after every 1000 entries. The winner will receive a Safeway Gift card for $500.

RMO Database: We will maintain a database for eligible individuals who refuse, are missed, or are otherwise excluded to assess the generalizability of our study sample**.**

Enrolment Criteria – (*Appendix D – Patient Screening Forms*) Children 12-60 months who present to one of the participating ED and have wheeze on auscultation. Those children who had oral antibiotics during the past 30 days, those whom physician consider to provide antibiotics, those with known hypersensitivity to macrolides, those with significant comoribiditites, those who are already enrolled in another study, those who does not communicate in English, those without telecommunication access, and those who are unable

to follow up in next 21 days will be excluded from the study. (*Appendix E – Detailed Inclusion and Exclusion Criteria)*

Baseline Assessment and Patient History*:* In addition to age, sex, postal code, vital signs and symptoms, a background questionnaire will be performed to document risk of multiple-trigger wheeze and asthma (i.e. wheezing/coughing in the past day and night, current and past medication usage in the last three months, other medical problems, atopic dermatitis, family history of atopy or asthma, parental smoking (*Appendix F – Patient Data Forms*).

Allocation, randomization, and blinding of study treatment (intervention):

Randomization method: A 2 X 2 variable blocked randomization code (ranging between 4 and 8 per block), with stratification by study site and first versus previous episode of wheeze, will be produced by the Drug Development and Innovation Centre at the university of Alberta (DDIC) using random-number generating software. DDIC will produce the study packages, complete the block randomization, and number the bottles. The blinded and numbered study drug packages, in addition to the master code will be available to both study sites if de-blinding is required. Stratification by 'history of wheeze' will allow us to track the number enrolled into each sub-group, and ensure we achieve our targeted sample size for each one. We have included children with both first and prior episode of wheeze and powered our study to perform a sub-group analysis for these two groups. Macrolide therapy has been shown to be effective in both bronchiolitis [57] and asthma [59]; incorporating both populations into this study design may provide important insights into how these populations of patients might differ.

Children will be defined as first-time wheezer if they have never had symptoms of wheeze prior to this episode, the symptoms of wheeze have been occurring for < 1 month with no period without resolution of symptoms for greater than 1 week during that 1 month period. Resolutions of symptoms include absence of all respiratory symptoms including wheeze, cough, respiratory distress, and shortness of breath. Some illustrative examples: If the patient has had symptoms for greater than 1 month- these children would be considered a repeat wheezer. If the child has had symptoms of wheezing for 1 month but had a 1 week period without respiratory symptoms in that one month, and we are seeing them after the 1 week resolution period, these children would be also be considered a repeat wheezer. IF the child has had wheezing symptoms for 1 month but was never symptoms free for more than 1 week continuously, this child would be considered a first-time wheezer.

Blinding of azithromycin: The pharmacy at each site will prepare two boxes (one for children enrolled with 'first episode of wheeze' and the other for those with 'prior episode of wheeze') of sequential patient packets containing the study drug in sealed, opaque, numbered bags. Each bag will contain 2 X 10 cc syringes, and either study drug or placebo that will be indistinguishable by appearance, smell, and taste. The two boxes of patient packets (first-time wheezing patient, previous wheezing patient box) will be kept in a locked and secure room in the ED for use as patients are enrolled. The research assistant who assigns the intervention will be unaware of the next group assignment as study drugs will be packaged identically and identified only by a sequential study number.

Randomization code access: The Research team from both sites will have access to the master randomization code until patient enrolment is completed and all decisions regarding statistical analyses are finalized.

Study Intervention: Children will be randomized to receive either azithromycin or placebo (powder to be produced by DDIC given at 10 mg/kg/day for day 1, then 5 mg/kg for 4 days. Each bottle will contain sufficient drug to adequately dose children who weigh up to 30 kg (>> 95%tile weight for 60 month old child). Excess study drug/placebo will be expelled from each syringe by the research assistant, so that the remaining volume of study drug/placebo will equal the appropriate dose based on the child's weight. An ED nurse will administer the first dose of the study drug/placebo. If the child vomits the first dose within 20 minutes of administration, a second dose will be given. The second syringe will be marked with the calculated dose level for days 2 to 5, labelled 'study drug', and provided to the parents at discharge from the ED. Parents will also be given a salbutamol metered dose inhaler (Airomir™, 100 μg per puff) fitted with a dose counter (Doser™, Meditracker), a spacer with mask (OptiChamber Advantage®, Philips Respironics), and advised to administer two puffs from the MDI via the spacer as needed, up to every four hours, if their child has symptoms of respiratory distress, wheeze, or persistent cough**.** Only children, who the attending ED feels would benefit from salbutamol at home, will have their salbutamol dispensed and their salbutamol usage tracked by Doser™ and by questionnaire. Children with wheeze on presentation who do not get prescribed salbutamol on discharge are still eligible for the study. As they were not prescribed salbutamol, their medication usage will not be tracked (secondary outcome). We will still track their primary outcome (time to resolution of symptoms)

Study Drug Compliance: Parents will also be asked to complete a daily medication diary (*Appendix G – Ordinal Daily Symptom and Drug Dosing Diary*) documenting administration of the study drug and to return the diary with the study drug bottle, MDI when they return for follow-up 21 days after enrolment. The medication diary will be reviewed and verified by the study coordinator at both telephone and in-person follow-up. If the child's respiratory symptoms are persistent for all 21 days of the calendar, a second calendar and a new MDI will be provided to the family.

Co-Interventions and Contamination: Staff ED physicians will be asked not to treat enrolled patients with antibiotics. Otherwise physicians will be allowed to prescribe any treatment, in addition to the study drug and rescue SABA, they judge to be necessary (e.g. oral or inhaled corticosteroids). While treatment with corticosteroids has the potential to contaminate the effects of the study medication, given our study's randomized design, each treatment group will most likely have comparable numbers of patients treated with co-interventions. All medications (including duration and dosages) patients receive at enrolment and during follow-up will be documented. A letter will also be sent to the child's primary care physician at the time of enrolment detailing the nature of the study and that the child has been randomized to either azithromycin or placebo to be taken for 5 days in total.

***Data gathering and Patient Follow-up** (Appendix H – Data quality & monitoring)*

<u>Daily Symptom Diary:</u> Parents will be given a Ordinal Daily Symptom Diary with the following ordered categories: heart = 'no respiratory symptoms'; *green star* = 'mild respiratory symptoms'; *yellow star* = 'respiratory symptoms are worse than usual or more medication than usual was required'; *red star* = 'respiratory symptoms sufficiently severe to require an unscheduled visit to a physician or treatment with oral corticosteroids' or Blue star = in addition to one of the above and a cold or flu symptoms appear. Star stickers are to be provided to place in blue boxes each day of study syrup is provided. Parents will be

instructed to focus on any noisy breathing respiratory symptom (wheezing or whistling sounds), breathlessness with eating or speaking, shortness of breath after activity or exposure to cold, or persistent troublesome coughing. (*Appendix G – Ordinal Daily Symptom and Drug Dosing Diary* [43]). This Ordinal Daily Symptom Dairy has been used in a prior study by PM [40] with excellent parental and child compliance, and good discrimination in respiratory symptoms between treatment groups (Montelukast versus placebo).

Telephone follow-up to day 21: Patients' parents will be contacted by a research assistant at 24 hours, 72 hours, 5 days, and 14 days following enrolment by telephone to remind them to take their study medication (up to the 5 day follow-up), complete their daily symptom and medication dairies, document if parents have returned to a physician for follow-up care for on-going symptoms, document on-going symptoms of respiratory distress, and, as a redundancy measure, to document collected data from the Daily Symptom and Drug Dosing Diary, and again prior to day 21 to remind the follow up visit at Day 21. Parent's will also be asked about the previous day's calendar stickers. (*Appendix I – Patient Telephone Follow-Up Form*). If patient experiences symptoms consistent with an adverse event, the study RA will contact the study co-ordinator who will contact the family, investigate the symptoms, and promptly provide the information to the site PI. Based on the results of the follow-up discussion, the site PI will determine what follow-up care is required and the site PI or delegate will contact the family and advice. If the research assistant is unable to contact the family on a particular day, the information will be collected on the following day.

In-Person follow-up at day 21: Study participants will be asked to return to the ACH or SCH for assessment by the site study coordinator 21 days after study enrolment. The majority of wheezy pre-school have returned to their baseline symptoms following a RTI after 21 days from initial presentation (DJ, unpublished data). Parent(s) will be asked to bring the Daily Symptom and Drug Dosing Dairy, the MDI and dose counter, and the empty Study Medication syringes. The site study coordinators (experienced pediatric nurses) will complete a history and physical examination and if the patient continues to have on-going respiratory symptoms, the parents will be referred, as appropriate, to their family physician, pediatrician, or the ACH or SCH emergency department for assessment and management

Atopy will be assessed using skin prick testing (SPT). Highly standardized ALK allergens will be used including tree, grass and weed pollens, cat, dog, *D. pteronyssinus*, *D. farinae*, cockroach, *Alternaria, Cladosporium, Aspergillus, and Penicillium.* Cow's milk, egg white, soy, wheat and peanut will also be tested. The same batch of each allergen will be used for all subjects. The wheal and flare, at 10 min to histamine and at 15 min for allergens, will be outlined in pen and transferred to paper (hard copy) by adhesive tape. The maximum weal and flare diameters and their mid-point perpendicular responses will be measured. Data will be corrected for any negative control response. Mean responses of both 2mm and 3mm greater than the negative control will be used in analyses, allowing comparison with studies using either of these wheal sizes to indicate a positive response.

Follow-up post day 21: After the follow-up visit at 21 days, a research assistant will contact the parents on day 35 by telephone. At this interview we will collect any adverse events and whether a repeat exacerbation has occurred. Specifically, the parent(s) will be asked if their children has been taken to a physician's office, urgent care clinic, or ED for treatment of worsening respiratory symptoms (*Appendix I – Patient Telephone Follow-Up Form day 35*). Follow-up will be either by phone or by email depending on the parent's preference.

<u>*Viral Testing*</u>*:* Nasopharyngeal swabs will be collected at the time of presentation to the ED and at 21 day follow-up, and tested for wide-range of viruses including human bocavirus, influenza A, influenza B, parainfluenza 1-4, RSV, adenoviruses including H1N1, human metapneumovirus, coronaviruses (OC43, 229E, NL63 and HKU1) and picornaviruses (rhinoviruses and enteroviruses) by utilizing molecular-based procedures.

<u>*Culture independent bacterial profiling:*</u> Nasopharyngeal swabs will be used for culture independent profiling of bacteria (*Appendix K – Details of Culture Independent Bacterial Profiling*). Briefly, total DNA will be prepared from all NP swabs obtained at enrolment and at day 21 follow-up. All samples will be analyzed by 16s rRNA gene amplification and Terminal Restriction Fragment Length Polymorphism (T-RFLP) analysis [65]. Forty of the initial presentation samples will also be analysed by in depth 16S rRNA gene sequencing using Flx Titanium Pyrosequencing to generate >150,000 gene sequences. This 16S rRNA gene library will be used to generate a pediatric nasopharyngeal microbiome database and *in silico* restriction fragment profile for T-RFLP peak identification. Comparison of presentation and post treatment samples will be used to identify specific bacterial responses to azithromycin treatment. We predict that post-treatment samples should a lower overall bacterial load but will have a T-RFLP profile that reflects the baseline normal microbiota for the nasopharynx. The T-RFLP profiles will also be examined for signatures corresponding to specific pathogens such as *Mycoplasma pneumoniae* and *Chlamydophila pneumoniae*. When present, these samples will be further characterized by quantitative PCR. The strategy outlined in Appendix K will capture atypical bacterial and pathogens that elude detection by standard clinical microbiology.

<u>*Medical* Chart *review*</u>: Each patient’s ACH, SCH, or other Alberta hospital medical records will be reviewed at 6 months following enrolment to confirm any visits to an emergency department, admissions to hospital, and document the diagnosis and treatment they received (*Appendix L – Medical Chart Review Form*). A random number of charts will be independently reviewed by both an investigator and a research assistant to establish reliability of data abstraction. Private physicians’ offices will also be contacted to confirm the dates and purpose of any visits.

<u>*SABA usage*</u>*:* As noted previously, the parents will be given a salbutamol MDI fitted with a dose counter (Doser™, Meditracker) to treat their child at discharge from the ED, and then collected at In-Person Follow-up on day 21. The Doser™ has the capability of logging without the user’s awareness the number of times the MDI is administered each day for up to 30 days. This will provide an objective count of the number of days an enrolled child receives rescue bronchodilator treatment.

Outcome Measurements*:*

<u>*Primary Outcome:*</u> - The primary outcome will be the time taken for children to resolve their acute respiratory symtoms (defined as three consecutive days of their child having either no (*green star*) or usual (*yellow star*) respiratory symptoms (*Appendix G – Ordinal Daily Symptom and Drug Dosing Diary*)). If a child at the 21 day follow-up has not resolved their acute symptoms, parents will be given another Nominal Daily Score Diary, and asked to continue to monitor symptoms for another 21 days at which time they will be asked to return for another in-person follow-up visit with the Site Study Coordinator. While it is extremely unlikely that any child’s acute respiratory symptoms will persist this long, this process will be repeated until the child’s acute respiratory symptoms resolve or they reach the end of follow-up at six months, whichever occurs first. We have chosen the number of days to

return to baseline respiratory symptoms as our primary outcome because persistent respiratory symptoms can have a major impact on a family's quality of life through missed sleep, school/day care, or work. Global assessment of respiratory symptoms by parents is often used as the primary outcome in this age of child because of the difficulties in obtaining more objective outcomes [87, 88].

Secondary Outcome: - Secondary outcomes will be 1) the number of days children are given a SABA in the 21 days following study enrolment, and 2) the time to disease exacerbation at 6 months follow-up. The number of days children are given SABA has been chosen as one of two secondary outcomes because it is an objective measure of disease activity, and should parallel and compliment the primary outcome. Only children prescribed a SABA at the time of discharge from the ED will be included in the analysis of the secondary outcome. The other secondary outcome, disease exacerbation, will be defined as an unscheduled visit to a physician/nurse practitioner or treatment with an oral corticosteroid for acute symptoms of cough, wheeze, or respiratory distress (based on parental perception) that occurs after the child's initial symptoms have resolved. Primary care physician chart extraction at 6 month follow-up will be used to identify any reported unscheduled physician visits for respiratory symptoms. We have chosen time to disease exacerbation as a secondary outcome because it will capture another potential way in which azithromycin may benefit the patient. If a child has clinically occult sinusitis, then resolution of this infection could remove the trigger for a child's recurrent episodes and allow a longer period free of respiratory symptoms.

Other Outcomes: - Additional outcomes will be the number of physician visits, missed day care/school by children, and missed hours of work for wages by either or both of the parents (hours missed per day*)* due to their child having cough, wheeze, or difficulty breathing.

Ethical considerations: Participation in this randomized placebo controlled clinical trial will involve nominal risk to the participants. There will be no significant inconvenience to families, no prolongation of their stay in the emergency department, no cost, and no significant pain, suffering, nor invasive tests. The study protocol will be reviewed and approved by the ethics review committee at each study site before the trial begins**.**

***Safety monitoring committee and termination policy**:* Adverse events will be actively monitored up to 30 days after the last of Azithromycin was given (day 5). Any child who develops symptoms consistent with adverse effects to azithromycin as evidenced by diarrhea or loose stools, nausea, abdominal pain, and vomiting (common side effects), symptoms of allergic reaction (hives), changes in mood or behaviour, arrhythmia/tachycardia will result in the review of their case by a safety monitoring committee consisting of a pediatric pulmonologist, allergist, and general pediatrician. The committee will review the case and decide whether to break the code and/or terminate the study based on the particulars of the case**.** If upon review there are concerns about the patient, the code would be broken and the randomization drug discontinued. The panel may consider terminating the study if there are profound adverse effects that would reflect concerns for all children.

Sample Size and Minimal Clinically Important Difference: The telithromycin in acute adult asthma study [60] demonstrated a 50% reduction in asthma symptoms among those using telithromycin (16% asthma symptoms in the placebo group versus 8% in the telithromycin group). We have used this data to estimate the magnitude of effect for our sample size calculations (a 50% decrease in the number of days to symptom resolution). Enrolment of 110 children per treatment arm per first-time versus previous wheeze (total 440), using a one-way ANOVA, will provide 80% power to detect at the .05 level, a 1.18 day

differences in the two treatment groups. This calculation assumes a conservative standard deviation of 6 days in symptoms duration [89] and a 10% loss to follow-up. For the planned sub-group analysis of first-time versus previous wheeze, we have sufficient power to detect a 1.36 day difference between the azithromycin and placebo groups (*Appendix M – Power Calculation*). We think that a reduction in the duration of symptoms by one day or more is likely to be considered meaningful because of its implication for a child missing daycare or school, or parents missing work.

Statistical Analysis: The baseline variables in the two groups will be compared overall and by enrolment center in terms of means, standard deviation, and range, and as appropriate, median, inter-quartile range, percentages and 95% confidence intervals. Imbalances between treatment groups for key baseline variables such as age, gender, duration of illness, presence of atypical bacteria, use of ICS or oral corticosteroids, and baseline clinical severity will indicate the need for an adjusted analysis. We will also include an interaction term in the analysis (drug X presence or atypical bacterial infection) to determine if azithromycin has a different degree of effectiveness among those with atypical bacteria present. Furthermore, for each response variable, we will perform an exploratory adjusted analysis incorporating enrolment center as a factor to ensure that unmeasured differences between enrolment centers do not bias our results. The primary outcome, time to symptom resolution, will be assessed for normality of distribution by the Wilk Shapiro Test and, if necessary, will be transformed to yield suitable distributions or Kruskal Wallis (non parametric ANOVA analogue) will be used to test the main hypothesis. If the one way ANOVA is significant, then, a-priori decided comparisons (placebo versus azithromycin in first-time wheezing children and placebo versus azithromycin in children with a prior wheeze history) will be tested via Tukey's Honest significant differences procedure. If an adjusted analysis is necessary, we will use an Analysis of Covariance model incorporating those baseline factors which are imbalanced between treatment groups and any other variable that is though to be of clinical relevance. An adjusted analysis will be performed in the event that co-intervention numbers (such as ICS or oral corticosteroid use) between the groups are not comparable. We also plan to perform a sub-group analysis to detect clinically significant differences in the each of the two wheezing sub-groups. Linear regression will be used to analyze β-agonist use during the 21 day follow-up period and Cox regression modelling will be used to analyse the time to next disease exacerbation.

7. QUALIFICATIONS / RESPONSIBILITIES OF INVESTIGATORS

This proposal will draw from the strengths in childhood respiratory care (pulmonary medicine, emergency medicine, infectious disease) and respiratory research (clinical epidemiology, microbiology, and basic mechanism of pulmonary disease) from across the province. Dr. David Johnson, MD, is a Pediatric Emergency physician and Professor with a 60% research appointment at the University of Calgary. He has led multicentre trials,[90-92] has expertise in the acute management of asthma,[93-96] and is past chair of Pediatric Emergency Research Canada, a national clinical research network [*http://perc.srv.ualberta.ca/]*. Dr. Piush Mandhane MD PhD, an Assistant Professor in Pediatric Pulmonology with a 75% research commitment at the University of Alberta, brings expertise in asthma clinical trials [43] and epidemiology of asthma [97-99].

The group will be co-lead by the department heads of the pediatric pulmonary divisions; Dr. C. Majaesic, MD, PhD at the University of Alberta and Dr. S Spier, MD, at the

University of Calgary. The responsibility of patient recruitment and follow-up will be coordinated by the site research coordinators and supervised by Drs. Johnson and Mandhane. We have secured the collaboration of a biostatistician, Alberto Nettel-Aguirre, PhD PStat, Research Methods Team, Department of Pediatrics, University of Calgary who has helped design the study and will work with us throughout the project. This study will also assess the atypical bacteria and viral etiology of wheezing in our children. As such Drs. Michael Surette (Professor, Department of Microbiology & Infectious Disease, University of Calgary), Bonita Lee (Associate Professor, Pediatric Infectious Diseases and Research Director for Virology in the Alberta), and James Kellner (Chair of Pediatrics, former Head of Pediatric Infectious Diseases, University of Calgary) are co-investigators on this project (*Appendix O – Common CVs of Investigators).*

8. ANTICIPATED RESULTS, TIMELINES, and KNOWLEDGE TRANSLATION

This study provides us an opportunity to test the use of antibiotics in wheezing children; a treatment strategy frequently used by community physicians [100]. A positive study will help us improve the management of this difficult to treat population. A negative study provides us an opportunity to educate community physicians on the lack of evidence for antibiotics in preschool children with wheeze.

Knowledge translation strategies, in addition to publication in peer-reviewed journals, will include incorporation of our findings into future versions of the Canadian Asthma Consensus Report, and an Alberta provincial clinical pathway for children with asthma. (Note these initiatives incorporate preschool children into their recommendations.) Incorporation of this evidence is assured as Dr. Spier is a long-serving member of the Canadian Asthma Consensus working group [101]and Dr. Johnson co-chairs and Drs. Spier and Majaesic are members of the Alberta Health Services (AHS) committee charged with developing and implementing a provincial childhood asthma pathway. The planned implementation strategy for the AHS pathway initiative is to develop a highly interactive web-curriculum module designed for primary care practitioners, and disseminate it through the 95 Alberta hospitals and 30 primary care networks to physicians, nurses, and respiratory therapists who care for Albertan children with asthma.

9. A PLATFORM FOR A CONTINUED UNDERSTANDING OF CHILDHOOD RESPIRATORY DISEASE

This project will allow the formation of the *Alberta Paediatric Research in Lung health (APRIL) Collaboration* whose goal will be to promote and enhance clinical and basic science research in pediatric pulmonary disease in Alberta. Future plans include an annual business meeting and scientific symposium as part of the Alberta Respiratory Disease Symposium (ARDS). Based on the results of this trial, future APRIL studies could include the role of macrolide antibiotics in older children with acute asthma exacerbations or completion of the three-arm RCT of ICS versus combination therapy (ICS + LABA) versus placebo as proposed in our original submission.

REFERENCES

1. Martinez FD, Wright AL, Taussig LM, Holberg CJ, Halonen M, Morgan WJ. Asthma and wheezing in the first six years of life. The Group Health Medical Associates. N Engl J Med 1995; 332:133-8.
2. Bisgaard H, Szefler S. Prevalence of asthma-like symptoms in young children. Pediatr Pulmonol 2007; 42:723-8.
3. Stevens CA, Turner D, Kuehni CE, Couriel JM, Silverman M. The economic impact of preschool asthma and wheeze. Eur Respir J 2003; 21:1000-6.
4. Frey U, von Mutius E. The challenge of managing wheezing in infants. N Engl J Med 2009; 360:2130-3.
5. Tamaoki J, Kadota J, Takizawa H. Clinical implications of the immunomodulatory effects of macrolides. Am J Med 2004; 117 Suppl 9A:5S-11S.
6. Parnham MJ. Immunomodulatory effects of antimicrobials in the therapy of respiratory tract infections. Curr Opin Infect Dis 2005; 18:125-31.
7. Gould IM. BTS guidelines on CAP. Community acquired pneumonia. Thorax 2002; 57:657.
8. Brand PL, Baraldi E, Bisgaard H, Boner AL, Castro-Rodriguez JA, Custovic A, et al. Definition, assessment and treatment of wheezing disorders in preschool children: an evidence-based approach. Eur Respir J 2008; 32:1096-110.
9. Doull IJ, Lampe FC, Smith S, Schreiber J, Freezer NJ, Holgate ST. Effect of inhaled corticosteroids on episodes of wheezing associated with viral infection in school age children: randomised double blind placebo controlled trial. Bmj 1997; 315:858-62.
10. McKean MC, Hewitt C, Lambert PC, Myint S, Silverman M. An adult model of exclusive viral wheeze: inflammation in the upper and lower respiratory tracts. Clin Exp Allergy 2003; 33:912-20.
11. Kurukulaaratchy RJ, Fenn MH, Waterhouse LM, Matthews SM, Holgate ST, Arshad SH. Characterization of wheezing phenotypes in the first 10 years of life. Clin Exp Allergy 2003; 33:573-8.
12. Lau S, Illi S, Sommerfeld C, Niggemann B, Volkel K, Madloch C, et al. Transient early wheeze is not associated with impaired lung function in 7-yr-old children. Eur Respir J 2003; 21:834-41.
13. Papadopoulos NG, Kalobatsou A. Respiratory viruses in childhood asthma. Curr Opin Allergy Clin Immunol 2007; 7:91-5.
14. Diagnosis and management of bronchiolitis. Pediatrics 2006; 118:1774-93.
15. Peroni DG, Piacentini GL, Ceravolo R, Boner AL. Difficult asthma: possible association with rhinosinusitis. Pediatr Allergy Immunol 2007; 18 Suppl 18:25-7.
16. Rachelefsky GS, Katz RM, Siegel SC. Chronic sinus disease with associated reactive airway disease in children. Pediatrics 1984; 73:526-9.
17. Bucca C, Rolla G, Scappaticci E, Chiampo F, Bugiani M, Magnano M, et al. Extrathoracic and intrathoracic airway responsiveness in sinusitis. J Allergy Clin Immunol 1995; 95:52-9.
18. Tsao CH, Chen LC, Yeh KW, Huang JL. Concomitant chronic sinusitis treatment in children with mild asthma: the effect on bronchial hyperresponsiveness. Chest 2003; 123:757-64.

19. Krawiec ME, Westcott JY, Chu HW, Balzar S, Trudeau JB, Schwartz LB, et al. Persistent wheezing in very young children is associated with lower respiratory inflammation. Am J Respir Crit Care Med 2001; 163:1338-43.
20. Milner AD. The role of corticosteroids in bronchiolitis and croup. Thorax 1997; 52:595-7.
21. Flores G, Horwitz RI. Efficacy of beta2-agonists in bronchiolitis: a reappraisal and meta-analysis. Pediatrics 1997; 100:233-9.
22. Baraldi E, Dario C, Ongaro R, Scollo M, Azzolin NM, Panza N, et al. Exhaled nitric oxide concentrations during treatment of wheezing exacerbation in infants and young children. Am J Respir Crit Care Med 1999; 159:1284-8.
23. Moeller A, Franklin P, Hall GL, Turner S, Straub D, Wildhaber JH, et al. Inhaled fluticasone dipropionate decreases levels of nitric oxide in recurrenty wheezy infants. Pediatr Pulmonol 2004; 38:250-5.
24. Straub DA, Moeller A, Minocchieri S, Hamacher J, Sennhauser FH, Hall GL, et al. The effect of montelukast on lung function and exhaled nitric oxide in infants with early childhood asthma. Eur Respir J 2005; 25:289-94.
25. Straub DA, Minocchieri S, Moeller A, Hamacher J, Wildhaber JH. The effect of montelukast on exhaled nitric oxide and lung function in asthmatic children 2 to 5 years old. Chest 2005; 127:509-14.
26. Holmgren D, Bjure J, Engstrom I, Sixt R, Sten G, Wennergren G. Transcutaneous blood gas monitoring during salbutamol inhalations in young children with acute asthmatic symptoms. Pediatr Pulmonol 1992; 14:75-9.
27. Kraemer R, Frey U, Sommer CW, Russi E. Short-term effect of albuterol, delivered via a new auxiliary device, in wheezy infants. Am Rev Respir Dis 1991; 144:347-51.
28. Conner WT, Dolovich MB, Frame RA, Newhouse MT. Reliable salbutamol administration in 6- to 36-month-old children by means of a metered dose inhaler and Aerochamber with mask. Pediatr Pulmonol 1989; 6:263-7.
29. Pool JB, Greenough A, Gleeson JG, Price JF. Inhaled bronchodilator treatment via the nebuhaler in young asthmatic patients. Arch Dis Child 1988; 63:288-91.
30. Nielsen KG, Bisgaard H. Bronchodilation and bronchoprotection in asthmatic preschool children from formoterol administered by mechanically actuated dry-powder inhaler and spacer. Am J Respir Crit Care Med 2001; 164:256-9.
31. Avital A, Godfrey S, Schachter J, Springer C. Protective effect of albuterol delivered via a spacer device (Babyhaler) against methacholine induced bronchoconstriction in young wheezy children. Pediatr Pulmonol 1994; 17:281-4.
32. Primhak RA, Smith CM, Yong SC, Wach R, Kurian M, Brown R, et al. The bronchoprotective effect of inhaled salmeterol in preschool children: a dose-ranging study. Eur Respir J 1999; 13:78-81.
33. Castro-Rodriguez JA, Rodrigo GJ. Efficacy of inhaled corticosteroids in infants and preschoolers with recurrent wheezing and asthma: a systematic review with meta-analysis. Pediatrics 2009; 123:e519-25.
34. Kaditis AG, Winnie G, Syrogiannopoulos GA. Anti-inflammatory pharmacotherapy for wheezing in preschool children. Pediatr Pulmonol 2007; 42:407-20.
35. Ducharme FM, Lemire C, Noya FJ, Davis GM, Alos N, Leblond H, et al. Preemptive use of high-dose fluticasone for virus-induced wheezing in young children. N Engl J Med 2009; 360:339-53.

36. McKean M, Ducharme F. Inhaled steroids for episodic viral wheeze of childhood. Cochrane Database Syst Rev 2000:CD001107.
37. Wilson N, Sloper K, Silverman M. Effect of continuous treatment with topical corticosteroid on episodic viral wheeze in preschool children. Arch Dis Child 1995; 72:317-20.
38. Panickar J, Lakhanpaul M, Lambert PC, Kenia P, Stephenson T, Smyth A, et al. Oral prednisolone for preschool children with acute virus-induced wheezing. N Engl J Med 2009; 360:329-38.
39. Vuillermin P, South M, Robertson C. Parent-initiated oral corticosteroid therapy for intermittent wheezing illnesses in children. Cochrane Database Syst Rev 2006; 3:CD005311.
40. Silverman M, Wang M, Hunter G, Taub N. Episodic viral wheeze in preschool children: effect of topical nasal corticosteroid prophylaxis. Thorax 2003; 58:431-4.
41. Bisgaard H, Zielen S, Garcia-Garcia ML, Johnston SL, Gilles L, Menten J, et al. Montelukast reduces asthma exacerbations in 2- to 5-year-old children with intermittent asthma. Am J Respir Crit Care Med 2005; 171:315-22.
42. Robertson CF, Price D, Henry R, Mellis C, Glasgow N, Fitzgerald D, et al. Short-course montelukast for intermittent asthma in children: a randomized controlled trial. Am J Respir Crit Care Med 2007; 175:323-9.
43. Johnston NW, Mandhane PJ, Dai J, Duncan JM, Greene JM, Lambert K, et al. Attenuation of the September epidemic of asthma exacerbations in children: a randomized, controlled trial of montelukast added to usual therapy. Pediatrics 2007; 120:e702-12.
44. Jaffe A, Bush A. Anti-inflammatory effects of macrolides in lung disease. Pediatr Pulmonol 2001; 31:464-73.
45. Tamaoki J, Takeyama K, Tagaya E, Konno K. Effect of clarithromycin on sputum production and its rheological properties in chronic respiratory tract infections. Antimicrob Agents Chemother 1995; 39:1688-90.
46. Takizawa H, Desaki M, Ohtoshi T, Kawasaki S, Kohyama T, Sato M, et al. Erythromycin modulates IL-8 expression in normal and inflamed human bronchial epithelial cells. Am J Respir Crit Care Med 1997; 156:266-71.
47. Desaki M, Okazaki H, Sunazuka T, Omura S, Yamamoto K, Takizawa H. Molecular mechanisms of anti-inflammatory action of erythromycin in human bronchial epithelial cells: possible role in the signaling pathway that regulates nuclear factor-kappaB activation. Antimicrob Agents Chemother 2004; 48:1581-5.
48. Kusano S, Kadota J, Kohno S, Iida K, Kawakami K, Morikawa T, et al. Effect of roxithromycin on peripheral neutrophil adhesion molecules in patients with chronic lower respiratory tract disease. Respiration 1995; 62:217-22.
49. Richeldi L, Ferrara G, Fabbri LM, Lasserson TJ, Gibson PG. Macrolides for chronic asthma. Cochrane Database Syst Rev 2005:CD002997.
50. Simpson JL, Powell H, Boyle MJ, Scott RJ, Gibson PG. Clarithromycin targets neutrophilic airway inflammation in refractory asthma. Am J Respir Crit Care Med 2008; 177:148-55.
51. Beigelman A, Gunsten S, Mikols CL, Vidavsky I, Cannon CL, Brody SL, et al. Azithromycin Attenuates Airway Inflammation in a Noninfectious Mouse Model of Allergic Asthma. Chest 2009.

52. Hashiba M, Baba S. Efficacy of long-term administration of clarithromycin in the treatment of intractable chronic sinusitis. Acta Otolaryngol Suppl 1996; 525:73-8.
53. Cervin A, Kalm O, Sandkull P, Lindberg S. One-year low-dose erythromycin treatment of persistent chronic sinusitis after sinus surgery: clinical outcome and effects on mucociliary parameters and nasal nitric oxide. Otolaryngol Head Neck Surg 2002; 126:481-9.
54. Kimura N, Nishioka K, Nishizaki K, Ogawa T, Naitou Y, Masuda Y. Clinical effect of low-dose, long-term roxithromycin chemotherapy in patients with chronic sinusitis. Acta Med Okayama 1997; 51:33-7.
55. Wallwork B, Coman W, Mackay-Sim A, Greiff L, Cervin A. A double-blind, randomized, placebo-controlled trial of macrolide in the treatment of chronic rhinosinusitis. Laryngoscope 2006; 116:189-93.
56. Ficnar B, Huzjak N, Oreskovic K, Matrapazovski M, Klinar I. Azithromycin: 3-day versus 5-day course in the treatment of respiratory tract infections in children. Croatian Azithromycin Study Group. J Chemother 1997; 9:38-43.
57. Tahan F, Ozcan A, Koc N. Clarithromycin in the treatment of RSV bronchiolitis: a double-blind, randomised, placebo-controlled trial. Eur Respir J 2007; 29:91-7.
58. Korppi M. Macrolides and bronchiolitis in infants. Eur Respir J 2007; 29:1283-4; author reply 4-5.
59. Fonseca-Aten M, Okada PJ, Bowlware KL, Chavez-Bueno S, Mejias A, Rios AM, et al. Effect of clarithromycin on cytokines and chemokines in children with an acute exacerbation of recurrent wheezing: a double-blind, randomized, placebo-controlled trial. Ann Allergy Asthma Immunol 2006; 97:457-63.
60. Johnston SL, Blasi F, Black PN, Martin RJ, Farrell DJ, Nieman RB. The effect of telithromycin in acute exacerbations of asthma. N Engl J Med 2006; 354:1589-600.
61. Biscione GL, Corne J, Chauhan AJ, Johnston SL. Increased frequency of detection of Chlamydophila pneumoniae in asthma. Eur Respir J 2004; 24:745-9.
62. Martin RJ, Kraft M, Chu HW, Berns EA, Cassell GH. A link between chronic asthma and chronic infection. J Allergy Clin Immunol 2001; 107:595-601.
63. Daian CM, Wolff AH, Bielory L. The role of atypical organisms in asthma. Allergy Asthma Proc 2000; 21:107-11.
64. Esposito S, Blasi F, Arosio C, Fioravanti L, Fagetti L, Droghetti R, et al. Importance of acute Mycoplasma pneumoniae and Chlamydia pneumoniae infections in children with wheezing. Eur Respir J 2000; 16:1142-6.
65. Sibley CD, Parkins MD, Rabin HR, Duan K, Norgaard JC, Surette MG. A polymicrobial perspective of pulmonary infections exposes an enigmatic pathogen in cystic fibrosis patients. Proc Natl Acad Sci U S A 2008; 105:15070-5.
66. Parkins MD, Sibley CD, Surette MG, Rabin HR. The Streptococcus milleri group--an unrecognized cause of disease in cystic fibrosis: a case series and literature review. Pediatr Pulmonol 2008; 43:490-7.
67. McComb JM, Campbell NP, Cleland J. Recurrent ventricular tachycardia associated with QT prolongation after mitral valve replacement and its association with intravenous administration of erythromycin. Am J Cardiol 1984; 54:922-3.
68. Malhotra-Kumar S, Lammens C, Coenen S, Van Herck K, Goossens H. Effect of azithromycin and clarithromycin therapy on pharyngeal carriage of macrolide-

resistant streptococci in healthy volunteers: a randomised, double-blind, placebo-controlled study. Lancet 2007; 369:482-90.
69. Lee AJ, Maddix DS. Rhabdomyolysis secondary to a drug interaction between simvastatin and clarithromycin. Ann Pharmacother 2001; 35:26-31.
70. Ray WA, Murray KT, Meredith S, Narasimhulu SS, Hall K, Stein CM. Oral erythromycin and the risk of sudden death from cardiac causes. N Engl J Med 2004; 351:1089-96.
71. Milberg P, Eckardt L, Bruns HJ, Biertz J, Ramtin S, Reinsch N, et al. Divergent proarrhythmic potential of macrolide antibiotics despite similar QT prolongation: fast phase 3 repolarization prevents early afterdepolarizations and torsade de pointes. J Pharmacol Exp Ther 2002; 303:218-25.
72. Samarendra P, Kumari S, Evans SJ, Sacchi TJ, Navarro V. QT prolongation associated with azithromycin/amiodarone combination. Pacing Clin Electrophysiol 2001; 24:1572-4.
73. Arellano-Rodrigo E, Garcia A, Mont L, Roque M. [Torsade de pointes and cardiorespiratory arrest induced by azithromycin in a patient with congenital long QT syndrome]. Med Clin (Barc) 2001; 117:118-9.
74. Russo V, Puzio G, Siniscalchi N. Azithromycin-induced QT prolongation in elderly patient. Acta Biomed 2006; 77:30-2.
75. Schoenenberger RA, Haefeli WE, Weiss P, Ritz RF. Association of intravenous erythromycin and potentially fatal ventricular tachycardia with Q-T prolongation (torsades de pointes). Bmj 1990; 300:1375-6.
76. Corrao G, Botteri E, Bagnardi V, Zambon A, Carobbio A, Falcone C, et al. Generating signals of drug-adverse effects from prescription databases and application to the risk of arrhythmia associated with antibacterials. Pharmacoepidemiol Drug Saf 2005; 14:31-40.
77. Owens RC, Jr. QT prolongation with antimicrobial agents: understanding the significance. Drugs 2004; 64:1091-124.
78. Harris S, Hilligoss DM, Colangelo PM, Eller M, Okerholm R. Azithromycin and terfenadine: lack of drug interaction. Clin Pharmacol Ther 1995; 58:310-5.
79. Shaffer D, Singer S, Korvick J, Honig P. Concomitant risk factors in reports of torsades de pointes associated with macrolide use: review of the United States Food and Drug Administration Adverse Event Reporting System. Clin Infect Dis 2002; 35:197-200.
80. Crosbie PA, Woodhead MA. Long-term macrolide therapy in chronic inflammatory airway diseases. Eur Respir J 2009; 33:171-81.
81. Tramper-Stranders GA, Wolfs TF, Fleer A, Kimpen JL, van der Ent CK. Maintenance azithromycin treatment in pediatric patients with cystic fibrosis: long-term outcomes related to macrolide resistance and pulmonary function. Pediatr Infect Dis J 2007; 26:8-12.
82. Phaff SJ, Tiddens HA, Verbrugh HA, Ott A. Macrolide resistance of Staphylococcus aureus and Haemophilus species associated with long-term azithromycin use in cystic fibrosis. J Antimicrob Chemother 2006; 57:741-6.
83. Reinert RR, Reinert S, van der Linden M, Cil MY, Al-Lahham A, Appelbaum P. Antimicrobial susceptibility of Streptococcus pneumoniae in eight European countries from 2001 to 2003. Antimicrob Agents Chemother 2005; 49:2903-13.

84. Malhotra-Kumar S, Lammens C, Chapelle S, Wijdooghe M, Piessens J, Van Herck K, et al. Macrolide- and telithromycin-resistant Streptococcus pyogenes, Belgium, 1999-2003. Emerg Infect Dis 2005; 11:939-42.
85. Livermore DM, Reynolds R, Stephens P, Duckworth G, Felmingham D, Johnson AP, et al. Trends in penicillin and macrolide resistance among pneumococci in the UK and the Republic of Ireland in relation to antibiotic sales to pharmacies and dispensing doctors. Int J Antimicrob Agents 2006; 28:273-9.
86. Swainston Harrison T, Keam SJ. Azithromycin extended release: a review of its use in the treatment of acute bacterial sinusitis and community-acquired pneumonia in the US. Drugs 2007; 67:773-92.
87. Bisgaard H, Hermansen MN, Loland L, Halkjaer LB, Buchvald F. Intermittent inhaled corticosteroids in infants with episodic wheezing. N Engl J Med 2006; 354:1998-2005.
88. Guilbert TW, Morgan WJ, Zeiger RS, Mauger DT, Boehmer SJ, Szefler SJ, et al. Long-term inhaled corticosteroids in preschool children at high risk for asthma. N Engl J Med 2006; 354:1985-97.
89. Heikkinen T, Jarvinen A. The common cold. Lancet 2003; 361:51-9.
90. Johnson DW, Jacobson S, Edney PC, Hadfield P, Mundy ME, Schuh S. A comparison of nebulized budesonide, intramuscular dexamethasone, and placebo for moderately severe croup. N Engl J Med 1998; 339:498-503.
91. Bjornson CL, Klassen TP, Williamson J, Brant R, Mitton C, Plint A, et al. A randomized trial of a single dose of oral dexamethasone for mild croup. N Engl J Med 2004; 351:1306-13.
92. Johnson DW, Craig W, Brant R, Mitton C, Svenson L, Klassen TP. A cluster randomized controlled trial comparing three methods of disseminating practice guidelines for children with croup [ISRCTN73394937]. Implement Sci 2006; 1:10.
93. Schuh S, Johnson DW, Stephens D, Callahan S, Winders P, Canny GJ. Comparison of albuterol delivered by a metered dose inhaler with spacer versus a nebulizer in children with mild acute asthma. J Pediatr 1999; 135:22-7.
94. Schuh S, Johnson DW, Callahan S, Canny G, Levison H. Efficacy of frequent nebulized ipratropium bromide added to frequent high-dose albuterol therapy in severe childhood asthma. J Pediatr 1995; 126:639-45.
95. Alotaibi S, Johnson D, Montegomery M, Sauve R, Spier S. Inhaled corticosteroids for abnormal pulmonary function in children with a history of chronic lung disease of infancy: study protocol [ISRCTN55153521]. BMC Pulm Med 2005; 5:6.
96. Pirie J, Cox P, Johnson D, Schuh S. Changes in treatment and outcomes of children receiving care in the intensive care unit for severe acute asthma. Pediatr Emerg Care 1998; 14:104-8.
97. Mandhane PJ, Greene JM, Cowan JO, Taylor DR, Sears MR. Sex differences in factors associated with childhood- and adolescent-onset wheeze. Am J Respir Crit Care Med 2005; 172:45-54.
98. Mandhane PJ, Greene JM, Sears MR. Interaction between breast-feeding, parental atopy, and sex on the development of asthma and atopy (abstract). JACI 2007; in press.

99. Mandhane PJ, Hanna SE, Inman M, Duncan JM, Greene JM, Hong H-Y, et al. Changes in exhaled nitric oxide related to estogen and progesterone during the menstrual cycle. CHEST 2009; In press.
100. Brand PL, van Aalderen WM. [Asthma in young children: a different approach to treatment by the family physician vs the pediatrician, a follow-up of symptoms over a period of 1 to 2.5 years]. Ned Tijdschr Geneeskd 1998; 142:1501-4.
101. Becker A, Lemiere C, Berube D, Boulet LP, Ducharme FM, FitzGerald M, et al. Summary of recommendations from the Canadian Asthma Consensus guidelines, 2003. Cmaj 2005; 173:S3-11.
